# Exploring the Mechanism of Total Flavonoids of Drynariae Rhizoma to Improve Large Bone Defects by Network Pharmacology and Experimental Assessment

**DOI:** 10.3389/fphar.2021.603734

**Published:** 2021-05-31

**Authors:** Weipeng Sun, Minying Li, Lei Xie, Zhexing Mai, Yan Zhang, Lieliang Luo, Zijian Yan, Zige Li, Hang Dong, Feng Huang, Zhen Shen, Ziwei Jiang

**Affiliations:** ^1^ The First School of Clinical Medicine, Guangzhou University of Chinese Medicine, Guangzhou, Guangdong Province, China; ^2^ Department of Orthopaedics, Kunming Municipal Hospital of Traditional Chinese Medicine, Kunming, Yunnan Province, China; ^3^ Department of Orthopaedics, The First Affiliated Hospital of Guangzhou University of Chinese Medicine, Guangzhou, Guangdong Province, China; ^4^ Medical College of Acupuncture-Moxibustion and Rehabilitation, Guangzhou University of Chinese Medicine, Guangzhou, Guangdong Province, China; ^5^ Science and Technology Innovation Center, Guangzhou University of Chinese Medicine, Guangzhou, Guangdong Province, China; ^6^ The Second School of Clinical Medicine, Guangzhou University of Chinese Medicine, Guangzhou, Guangdong Province, China

**Keywords:** drynariae rhizoma, experimental assessment, gusuibu, large bone defects, network pharmacology

## Abstract

Drynariae Rhizoma (DR) has been demonstrated to be effective in promoting fracture healing in clinical use. In the study, we tried to predicate potential signaling pathways and active ingredients of DR *via* network pharmacology, uncover its regulation mechanism to improve large bone defects by *in vivo* and *in vitro* experiment. We total discovered 18 potential active ingredients such as flavonoids and 81 corresponding targets, in which mitogen-activated protein kinase (MAPK) signaling pathway has the highest correlation with bone defects in pathway and functional enrichment analysis. Therefore, we hypothesized that flavonoids in DR improve large bone defects by activating MAPK signaling pathway. Animal experiments were carried out and all rats randomly divided into TFDR low, medium, and high dosage group, model group and control group. 12 weeks after treatment, according to X-ray and Micro-CT, TFDR medium dosage group significantly promote new bone mineralization compared with other groups. The results of HE and Masson staining and in vitro ALP level of BMSC also demonstrated the formation of bone matrix and mineralization in the TFDR groups. Also, angiographic imaging suggested that flavonoids in DR promoting angiogenesis in the defect area. Consistently, TFDR significantly enhanced the expression of BMP-2, RUNX-2, VEGF, HIF-1 in large bone defect rats based on ELISA and Real-Time PCR. Overall, we not only discover the active ingredients of DR in this study, but also explained how flavonoids in DR regulating MAPK signaling pathway to improve large bone defects.

## Introduction

Large bone defects (LBDs) are commonly caused by factors such as high-energy traumas, infections, tumors or congenital malformations, which are important reasons for loss of limb function and seriously affecting quality of life (Mediouni and Volosnikov, 2012) It is reported that LBDs affect over two million people worldwide with an economic burden of $3 billion every year(Bone Grafts and Substitutes - Global Analysis and Market Forecasts, 2014; Bone Graft Substitutes Market Report - Global Forecast to 2027, 2019). Distraction osteogenesis (DO) was established by Dr. Ilizarov (Gubin et al., 2016) and the basic principle of DO technique includes performing a transverse bone section (osteotomy) before gradually distracting the two bone segments. New bone tissue is generated in the gap between the two bone segments that are gradually and progressively distracted. DO is now a key surgical technique widely used in adult orthopedic surgery for various pathological conditions (Sailhan, 2011). Since the introduction of DO, its unique effectiveness on osteogenesis are significant compared with traditional techniques. However, DO also exists inevitable limitations. The slow calcification and formation of new bone as well as long clinical course are the biggest problem. How to effectively promote the formation of new bone, improve the quality of calcification and shorten the fixation time are burning issues for DO (Spiegl et al., 2013). FDA have approved BMP-2, BMP-7, and parathyroid hormone (PTH) to promote the effect of DO. However, due to the high cost, the inconvenience to carry, unstableness and other disadvantages, the results are not satisfying as expected in practical application.

Traditional Chinese medicine (TCM), as an important component of complementary and alternative medicine system, has been used to cure disease in China for over two thousand years (Tang et al., 2008). Several TCM formulas and herbal extracts have been proved to be effective for LBDs by promoting the formation of new bone and improving the quality of calcification (Hu et al., 2020).

Drynariae Rhizoma (DR) derived from Drynaria roosii Nakaike (Polypodiaceae) or Gusuibu is a classic Chinese herbal medicine contains mainly of flavonoids (flavanone) (Cao et al., 2017) and is prepared based on the standard of the China Pharmacopoeia standard of quality control. DR has been used in clinic as nourishing the kidneys, strengthening the bones, curing injuries and relieving pains (Zhang et al., 2017). What’s more, our previous studies have revealed that total flavonoids from Drynariae Rhizoma (TFDR) could promote fracture healing and the mechanism is mainly delineated by improving blood circulation, relieving blood flow abnormalities and preventing blood clots (Liu et al., 2015). However, whether TFDR exerts positive influences for LBDs and the potential mechanisms have not been elucidated.

Traditional Chinese herbs include multi-components and multi-targets, making it’s difficult to analyze by conventional experimental methods. Moreover, the clinical application of TCM in the world has been hampered because of unclear effects and mechanisms. It is thus necessary to clarify the scientific basis and potential mechanisms of Chinese herbs based on new approach.

Network pharmacology, combined with pharmacology and pharmacodynamics, is a novel research field which clarifies the synergistic effects and the underlying mechanisms of numerous compounds by analyzing various networks of the complex and multi-levels interactions (Zhang et al., 2015). Since network description and analysis deliver a system-level understanding of drug action and disease complexity, the system-pharmacological model will be of benefit for dissecting the functions of herbal medicines on special diseases (Zhang et al., 2014).

Therefore, this study will clarify the potential signaling pathway most related to bone defects and most active ingredients in DR based on network pharmacology before carried out experiments to uncover the underlying mechanism how DR activated the signaling pathway to improving LBDs.

## Materials and Methods

### Potential Components and Targets Prediction of Network Pharmacology

#### Chemical Candidates of TFDR

The chemical candidates of each ingredient in Drynariae Rhizoma were gathered from TCM systems pharmacology database (TCMSP, http://ibts.hkbu.edu.hk/LSP/tcmsp.php), (Zhang et al., 2018) TCM Database@Taiwan (http://tcm.cmu.edu.tw/) (Chen, 2011) and relevant literatures. A total of 18 components were collected.

#### Physicochemical Characteristics of Compounds in DR

The physicochemical properties of these compounds include molecular weight (MW), liquid-water partition coefficient (AlogP), the number of donor atoms for H-bonds (Hdon), the number of acceptor atoms for H-bonds (HACC), drug-likeness (DL) and oral bioavailability (OB). The characteristics of compounds in TFDR were all obtained from TCMSP database and the variables of basic properties in each compounds of DR were analyzed next.

#### Prediction of Protein Targets in DR

Protein targets are the fundamental ingredients of biology pathways and predicting the targets are actually helpful for clarifying the therapeutic mechanism of DR. The planar structure of each candidate was obtained from Pubchem database (https://pubchem.ncbi.nlm.nih.gov/). Swiss Target Prediction (http://www.swisstargetprediction.ch/), a web tool, provides prediction for the most possible protein targets of chemical compositions *via* reserve screening based on the similarity theory (Daina et al., 2019). The prediction will be performed when uploading the 2D structure of composition to this web tool. Finally, we gained 92 protein targets in total.

#### Related Targets of Osteogenesis in LBDs

The associated targets were collected from three databases, including 1) GeneCards database (https://www.genecards.org/), which is a searchable, integrative database that provides comprehensive, user-friendly information on all annotated and predicted human genes. We collected 1,737 genes related to osteogenesis in large bone defects from the database. 2) The Online Mendelian Inheritance in Man (OMIM) database (https://www.omim.org/). It is a comprehensive, authoritative compendium of human genes and genetic phenotypes that is freely available and updated daily. We searched the OMIM database with a keyword “osteogenesis” and found 144 genes. 3) The National Center for Biotechnology Information’s (NCBI) Gene database (https://www.ncbi.nlm.nih.gov/gene/), and 515 genes were found in NCBI Gene database. Finally, we totally uncovered 1,748 targets linked with osteogenesis after deleting redundancy.

#### Collection of Potential Therapeutic Targets and Network Construction

The Uniprot IDs for related targets of osteogenesis were taken from the Universal Protein Resource (UniProt) (https://www.uniprot.org/) by transforming the gene symbols of different databases into Uniprot ID (The UniProt Consortium, 2017). We compared targets in DR and targets of osteogenesis by contrasting their Uniprot IDs, and we selected the unduplicated part as potential therapeutic targets. A total of 81 potential therapeutic targets were harvested.

The correlations between chemical candidates and potential therapeutic targets were visualized by using Cytoscape 3.7.1 to conduct a pharmacological network.

#### Pathway Analysis

KOBAS 3.0, a web server, integrates five pathway databases (KEGG PATHWAY, PID, BioCyc, Reactome and Panther) and five disease databases (OMIM, KEGG DISEASE, FunDO, GAD and NHGRI GWAS Catalog), supplying functional annotation of gene or protein and enrichment of functional gene set. We performed the pathway and function analysis of the potential therapeutic targets *via* KOBAS 3.0. The pathways with applicable thresholds of *p* < 0.05 were reserved.

### Animals and Treatment

#### Experimental Animals

Animal experiments in the study were approved by the Institutional Animal Care and Use Committee of the First Affiliated Hospital of Guangzhou University of Chinese Medicine, China (No. 20190306032). A total of 120 SPF male Sprague-Dawley rats (age of 10–12 weeks, weight 280–320 g) were purchased from experimental animal center of Guangzhou University of Chinese Medicine and maintained at a room with constant temperature 23 ± 2°C, 12 h light/dark cycle, and free access to standard diet and water.

##### TFDR Preparation

In the network pharmacology study, researchers have confirmed that TFDR was the most effective ingredients of DR. Therefore, in this study, we applied Qianggu capsules as experimental drugs *in vivo*. Qianggu capsules were provided by the First Affiliated Hospital of Guangzhou University of Chinese Medicine, Guangdong, China. TFDR is an effective ingredient of Qianggu Capsules (produced by Beijing Qihuang Pharmaceutical Co., Ltd., batch number: 018304, net weight:0.25 g, each capsule contains 0.18 g TFDR).

#### Rats’ Distraction Osteogenesis Model Construction

A self-made patented Annular External Fixation Device and 3% Pentobarbital sodium (1.5 ml/kg) were used for intraperitoneal injection anesthesia in rats. After the anesthesia was in effect, rats’ right tibiae were shaved and prepared, and the surgical area was repeatedly sterilized with 75% alcohol. 80,000 units of gentamicin diluted with 250 ml of normal saline for lavage were prepared. Rat’s right knee joint and ankle joint was gently held the with both hands of researchers. Then, took the apex of the tibia as the body surface mark, and made a 1 cm longitudinal incision, applying blunt separation to superficial fascia. Researcher deeply detected the fibula that behind the tibia along the intermuscular space, cut the fibula with scissors and silk sutured the incision skin. The first ring-shaped tablet was placed. A 0.5 mm diameter Kirschner wire was drilled with an electric drill. The anterior medial portion of the upper tibia was inserted at 45° perpendicular to the tibial axis. Through the same method, used the second Kirschner wire to make an 30°–45°angle with the first coronal plane, and penetrated near the point of the first Kirschner wire. In the next place, placed the second ring-shaped tablet, and the two tablets and the crossed Kirschner wires between them were fixed with short screws. The position was adjusted so that the central axis of the calf of the rat coincided with the central axis of the circular tablet. Moreover, placed the third ring-shaped tablet, applied the above method inserting two Kirschner wires in the middle tibia, and placed the fourth tablet, using long screws to link the fixed tablets near and far. Finally, after the fixation was firmly fixed, removed the incision of the front end of the tibia, and re-exposed the tibia. A 1 mm drill bit was drilled through the midpoint of the tibia (while gentamicin was used for lavage and cooling). Surgeon cut the sieve-shaped tibia cut and measured the length of the osteotomy for 4 mm, trimmed the cortical edge, tightening the long screw nut. Research assistant flushed, sutured, and sterilized the surgical incision after the fixation was satisfied. To prevent infection, Penicillin was injected intramuscularly. The dosage was 40,000 units per day for three consecutive days. The medication time would be appropriately increased according to the wound healing condition, usually under 7 days. Fasting for 24 h, each rat was raised in a single cage until the lower limbs swelling disappeared within 7 days after the operation.

#### Treatment

After successful establishment of large tibial defects model, 120 rats were divided into five groups including: 1) TFDR low dosage group (CEF rats, *n* = 24); 2) TFDR medium dosage group (CEF rats, *n* = 24); 3) TFDR high dosage group (CEF rats, *n* = 24); 4) Model group (CEF rats, *n* = 24); 5) Control group (sham rats, *n* = 24). Low, medium and high-dose TFDR groups were given Qianggu Capsules solution at an equal volume for 12 weeks. Qianggu Capsules will be added into distilled water to make a certain concentration solution, and calculated the equivalent dose according to the body surface area. These three groups were given 0.11, 0.22, and 0.44 g⋅kg^−1^⋅d^−1^ gavage, weighed once a week during the experiment, and adjusted the dose according to weight. The model group and the blank group were given the same amount of saline for gavage.

#### Angiography

Three rats were randomly selected from each group at the fourth week after surgery. After anesthesia, the thorax was dissected and the venipuncture needle was inserted into the left ventricle. Rats were poured with heparin sodium saline (500 ml 0.9% physiological saline contained 100 Uml-1 heparin sodium), while 10% neutral buffered formalin was perfusion for tissue fixation. After that, heparin sodium saline was poured again into the rats, silicone rubber injection compound (Microfil MV-122, Flow Tech) was fully perfused.

#### X-Ray Examination

At the 12th week after surgery, general anesthesia was performed to each rat, and an X-ray machine was used to obtain X-ray images of the right tibia and fibula. The Lane-Sandhu X-ray score table was applied to evaluate the bone reconstruction level in the distraction area (Table 1).

**TABLE 1:** Lane-sandhu X-ray scoring.

Points	
Bone formation	
No evidence of bone formation	**0**
Bone formation occupying 25% of defect	**1**
Bone formation occupying 50% of defect	**2**
Bone formation occupying 75% of defect	**3**
Full gap bone formation	**4**
Union	
Full fracture line	**0**
Partial fracture line	**2**
Absent fracture line	**4**
Remodeling	
No evidence of remodeling	**0**
Remodeling of intramedullary canal	**2**
Full remodeling of cortex	**4**

#### Micro-CT Examination

Micro-CT examination was performed on the specimens after X-ray examination to analyze the parameters of bone histomorphometry. The specimen were placed in the detection tube of the Micro-CT system and scanned from top to bottom along the long axis of the tibia to obtain a connected Micro-CT image with an image resolution of 1,024 × 1,024, a pixel size of 36 × 36 μm, and a gray image Degree level 16 bit, layer spacing 27.37 μm, manual and semi-automatic selection of ROI, measurement and analysis of bone mineral density, bone volume fraction, bone mineral volume, bone surface area to bone volume ratio, structural model index, bone trabecular thickness, number of trabecular bone, trabecular bone clearance.

#### Histomorphology Assay

The tibia specimens obtained at the 12th week after surgery were fixed in 4% paraformaldehyde for 48 h, decalcified with 10% ethylenediaminetetraacetic acid (EDTA) for 21 days, embedded in paraffin, and a tissue microtome was used to longitudinally cut 4 μm slice that contained the backbone and callus. The morphological structure of the distraction area that observed stained with hematoxylin-eosin (HE) or Masson trichrome dye, and the bone reconstruction level in the bone defect area was evaluated.

### *In Vitro* Experiment

#### Preparation of TFDR-Containing Serum

The decoction of TFDR and boiled water was concentrated, stored at 4°C and returned to room temperature before medication. Twenty SPF male Sprague-Dawley rats were randomly divided into four groups, including the control group, TFDR low dosage group, TFDR medium dosage group, and TFDR high dosage group, with five rats in each group. All rats were gavaged at 8 a.m. and 2 p.m. per day for three consecutive days. The treatment groups were gavaged with different dosage of TFDR, 0.11, 0.22, and 0.44 g·kg^−1^·d^−1^, respectively. The control group was administered with equal volume of physiological saline. The blood from the abdominal aorta was collected 1.5 h after the last administration, then stood for 2 h and centrifuged with the speed of 2,500 r·min-1 at 4°C for 15 min to remove the upper serum. The serum was immersed in the 56°C water bathe to inactivate complements, filtered with 0.22 μm micropore filtration and preserved at −20°C.

#### Grouping and Administration

The bone marrow mesenchymal stem cells (BMSCs) were divided into the control group, TFDR low dosage group, TFDR medium dosage group, and TFDR high dosage group. The control group was given L-DMEM, 100 kU·L−1 streptomycin, 100 kU·L-1 penicillin, and 10% serum-containing medium. The other three groups were given L-DMEM, osteogenetic differentiation inducer, and the corresponding dosage of TFDR-containing serum.

#### Separation and Amplification of BMSCs

The Sprague-Dawley rats were anesthetized and soaked in 75% alcohol for 15 min. The surrounding tissues were removed rapidly, the tibial bone was separated and soaked in 75% alcohol for 30 s. The bone biting forcep was used to remove the epiphysis, and the L-DMEM medium containing 10% fetal calf serum, 100 kU·L−1 streptomycin as well as 100 kU·L−1 penicillin was sucked to wash the medullary cavity repeatedly until the osseous substance appearing slightly bright. The washing fluid of the medullary cavity was collected and transferred to a 25 cm two culture bottles. After culturing in a 5% CO2 incubator at 37°C for 24 h, then liquid exchange was performed for the first time and afterward every 2 days. When cell fusion reached over 80%, 0.25% trypsin was used to digest. Then the cells were subcultured at a ratio of 1:3. The well-grown cells of the third generation were selected to detect cell surface antigen with flow cytometry, thus immunophenotype of BMSCs was determined.

#### Identification of BMSCs

The flow cytometry was adopted to detect the expression of surface markers of BMSCs. The third generation of BMSCs were all blown into single cell suspension, transferred to the 10 ml centrifuge tube (1,000 r·min ^−1^, 5 min). After removal of the supernatant, the cells were washed with PBS, centrifuged repeatedly, and continuously washed for three times. The BMSCs concentration was adjusted to 2 × 109 L^−1^ and filtered with 200-mesh screen cloth. Four special tubes of flow cytometry were chosen, with 100 μlcell suspension, 5 μl CD34-PE, 5 μl CD44-APC mouse anti-human monoclonal antibody as well as 5 μl isotype antibody added in. The suspension was incubated for 15 min, and the flow cytometry was applied for statistical analysis.

#### Cell Viability Assay

The third generation of good growth of BMSCs in each group were selected. The single cell suspension was prepared after digested by trypsin, and then planted into the 12-well plates at a density of 1 × 107 L^−1^. Each well was added with 100 μlcell suspension containing about 1,000 cells. There were six holes in each group with corresponding medium added in, which were labeled and incubated in a 5% CO2 incubator at 37°C. The BMSCs exposed for 3, 5, 7, and 9 days were detected, respectively. The 10 μl Cell Counting Kit 8 (CCK-8) reagent was supplied to each hole and cell culture was terminated after incubation at 37°C for 4 h. The absorbance (A) of each hole was measured at 490 nm measured by microplate reader, therefore A490 reflected the cell viability level.

#### Alkaline Phosphatase Activity

Other third generation of BMSCs in each group were seeded into the 12-well plates at a density of 1 × 107 L−1 and the corresponding concentration of medium was added in. ALP activity was detected when BMSCs were consecutively cultured for 3, 10, and 13 days, respectively. The absorbance at 562 nm was also assessed. The third generation of BMSCs in each group were planted into the 12-well plates. When cells of each group were cultured for 21 days, the culture medium was discarded. After PBS washing, cells were fixed with 95% alcohol for 30 min, stained with 0.2% alizarin red for 30 min and rinsed with water. Then the cells were observed with a microscope and images were captured under 40 × microscopy. Twelve fields of vision were randomly selected to calculate the number of mineralized nodes in each group, indicating the mineralization ability of the BMSCs.

#### Quantitative Real-time Polymerase Chain Reaction (qRT-PCR)

The BMSCs were seeded into the 12-well plates at a density of 1 × 107L-1. When the bottom of the bottle was covered with more than 80% cells, the corresponding culture medium was added in each group. The liquid was exchanged every 2 days. After culturing BMSCs for 7 days, the total RNA of each group was extracted using the Trizol reagent. Ranged from 1.8 to 2.2, A260/A280 was tested by ultraviolet spectrophotometer to determine the purity of RNA. The reaction conditions of qPCR were as follows: predenaturation at 95°C for 5 min, a total of 40 circles of 95°C for 20 s, deformation at 62°C for 15 s, annealing at 72°C for 15 s, extension at 72°C for 5 min. The mRNA expression of each group was assessed, and the fluorescence amplification curves, standard curves as well as melting curves were drawn after reaction. The number of copy genes of each group was calculated based on the standard curves of target genes and the CT value was recorded. Relative expression of BMP-2 mRNA, p38 MAPK mRNA, VEGF mRNA, RUNX-2 mRNA, and HIF-1α mRNA was calculated according to the 2−ΔΔCt method. Repeated the test three times on each sample and the test primers were shown in Table 2.

**TABLE 2:** Sequences of primers.

Gene	Primer sequences (5′–3′)	
HIF-1α	Forward	ACA​AGA​AAC​CGC​CTA​TGA​CG
	Reverse	TAA​ATT​GAA​CGG​CCC​AAA​AG
VEGF	Forward	GTC​GGA​GAG​CAA​CGT​CAC​TA
	Reverse	TGC​GCT​TTC​GTT​TTT​GAC​CC
RUNX2	Forward	GCT​TGA​TGA​CTC​TAA​ACC​TA
	Reverse	AAA​AAG​GGC​CCA​GTT​CTG​AA
BMP2	Forward	CAG​CGA​GTT​TGA​GTT​GAG​G
	Reverse	CGG​TAC​AGG​TCG​AGC​ATA​T
β-actin	Forward	ATA​TCG​CTG​CGC​TGG​TCG​TC
	Reverse	AGG​ATG​GCG​TGA​GGG​AGA​GC

#### Western Blot Analysis

The protein expression of BMP-2, p38 MAPK, phosphorylation of p38 MAPK (p-p38 MAPK), VEGF, and RUNX-2 in each group was detected by specific antibodies using western blot analysis. Proteins from BMSCs were lyzzed with RIPA lysate. After the samples were centrifuged (12,000 r·min-1, 10 min, 4°C), the supernatant was extracted. The protein concentration was tested using a BCA protein assay kit. The protein sample (50 μg) was separated by SDS-PAGE, transferred to a PVDF membranes. The membranes were blocked with nonfat dry milk for 1 h. After incubation with antibodies (1:1,000, BMP-2, p38 MAPK, p-p38 MAPK, VEGF, RUNX-2, and HIF-1α) at 4°C overnight, the corresponding secondary antibodies (1:5,000) were added in and incubated at room temperature for 1 h. After displaying color by ECL kit, the gray values of protein strips were determined by the SensiAnsys image analysis software. The beta-actin was served as internal reference to compare the relative expression of proteins in each group.

### Statistical Analysis

Statistical analysis was carried out by SPSS 19.0. Two-way analysis of variance was applied to measure the significance of comparisons between groups after the homogeneity of variance was confirmed. Fisher’s Least Significant Difference test was utilized for comparative analysis between control group and other groups. Differences were considered statistically significant when p-value was less than 0.05. All quantitative data are shown as mean ± SEM.

## Results

### Ingredients Qualities in DR

**FIGURE 1:**
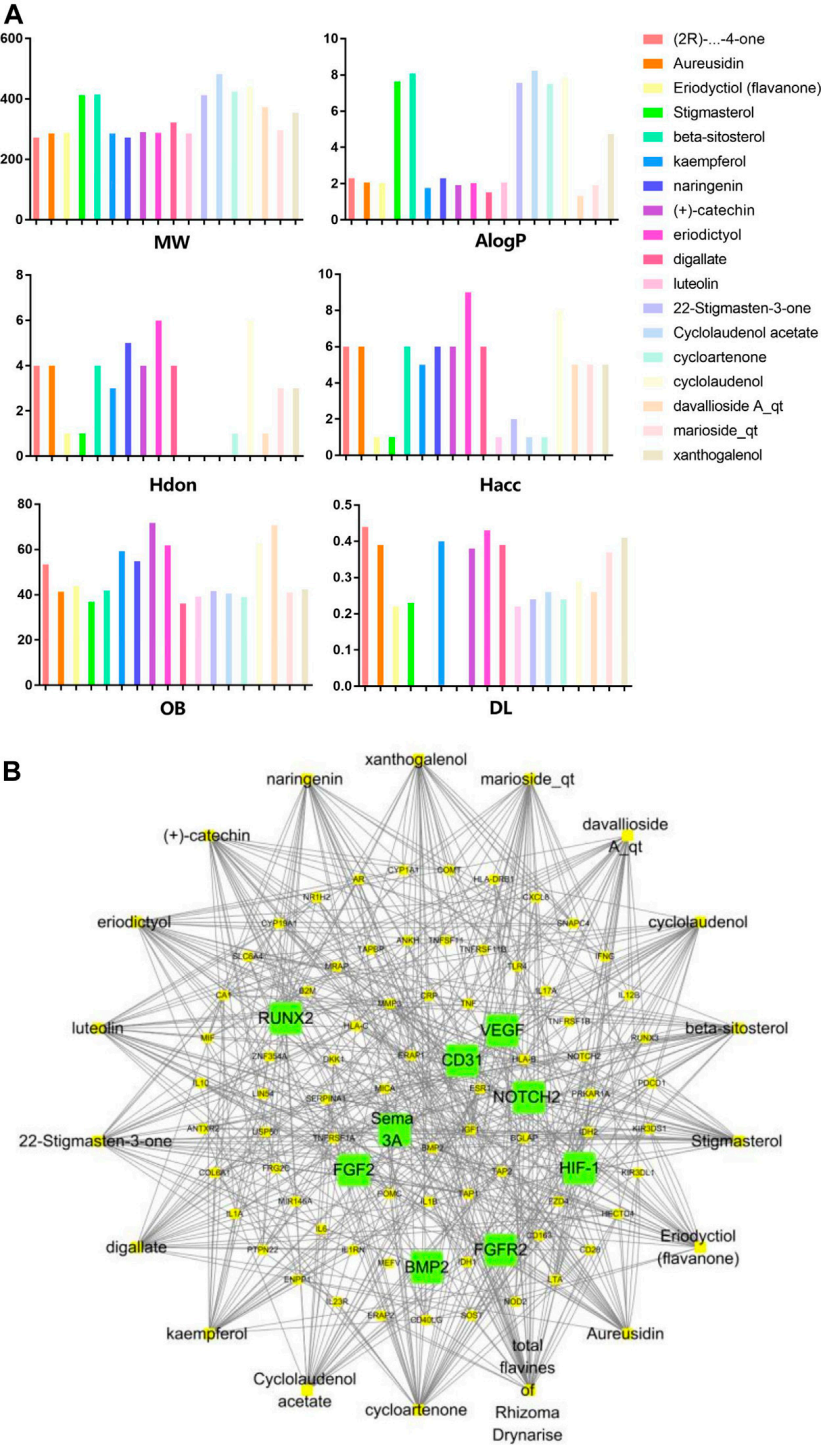
**(A)** The molecular properties of chemical candidates in RD. The 18 gathered chemical candidates were listed in the right side of the figure (2R)-...-4-one is the contraction of (2R)-5, 7-dihydroxy-2-(4-hydroxyphenyl) chroman-4-one; **(B)** chemical candidates-potential therapeutic targets network of RD acting on osteogensis. In the network, orange points represent the chemical candidates of RD, yellow points represent the therapeutic targets with weak linkage to osteogenesis and the huge green points stood for the core targets, being defined based on existing studies and data.

In order to explore in-depth about these ingredients, we compared six parameters including MW, ALogP, Hdon, Hacc, OB and DL, and all results were shown in Figure 1A. For the MW part, the values of all candidates were not only greater than 180 but less than 500 Dalton, indicating that all candidates were more druggable. For the ALogP part, most of the ingredients had low level of ALogP, except for *Stigmasterol*, *beta-sitosterol*, *22-Stigmasten-3-one*, *Cyclolaudenol acetate*, *cycloartenone and cyclolaudenol*, which may suggest the major ingredients of Drynariae Rhizoma had a great water solubility. For the aspects of Hdon and Hacc, *Eriodyctiol(flavanone)*, *Stigmasterol*, *luteolin*, *22-Stigmasten-3-one*, *Cyclolaudenol acetate*, *cycloartenone* were obviously different from other components because of less values of Hdon and Hacc. Besides, Hdon value of *davallioside A_qt* was also small. For the OB portion, we noticed that the values of whole compositions were greater than 30, declaring that they were easily absorbed by the body. In terms of DL, all but beta-sitosterol and naringenin owned the DL values greater than 0.18, which suggested that these components had a significant “drug-like” peculiarity(Li et al., 2020). In brief, though a small fraction of ingredients were unmatched to “drug-like” criteria, DR still had a promising potential of being an oral drug.

### Network Construction and Analysis

As described in the methods section, we carried out a “candidates-targets” network (Figure 1B; Table 3). The network was consisted of 18 candidate points (Table 4), 81 target points and 508 lines. In the network, the orange points represented the candidates, the yellow points presented the therapeutic targets with weak linkage to osteogenesis and the huge green points stood for the core targets, being defined based on existing studies and data. Runt-related transcription factor 2 (RUNX-2), vascular endothelial grow factor (VEGF), fibroblast growth factor receptor 2 (FGFR2) and CD31 (platelet/endothelial cell adhesion molecule) had biological function of coupling osteogenic differentiation with angiogenesis(Kawane et al., 2018; Qiao et al., 2018; Jiang et al., 2019). Semaphorin 3A played an essential role in regulating the osteogenic differentiation to promote bone regeneration(Zhang et al., 2018). High expressive fibroblast growth factor 2 (FGF2) could effectively enhance the process of vascular regeneration that was beneficial to bone repairation(Zhang et al., 2019). Additionally, the physiological function of Hypoxia-inducible factor 1 (HIF-1) was similar to FGF2(Huang et al., 2019). Bone morphogenetic protein 2 (BMP-2) was always considered as the key factor of osteogenesis. NOTCH2 participated in inhibiting osteogenesis caused by hyperglycemia(Ru et al., 2014).

**TABLE 3:** The core targets and their network degrees and related pathways.

Target	Description	Degree	Involved in pathway
VEGF	Vascular endothelial grow factor	45	MAPK, PI3K-Akt, VEGF signaling pathway
FGF2	Fibroblast growth factor 2	38	MAPK, PI3K-Akt, RANKL signaling pathway
RUNX2	Runt-related transcription factor 2	34	FoxO, NK-kappB signaling pathway
BMP2	Bone morphogenetic protein 2	30	TGF-beta, RANKL, BMP2 signaling pathway
NOTCH2	Neurogenic locus notch homolog protein 2	27	Notch signaling pathway
Sema 3A	Semaphorin 3A	23	RANKL, T cell receptor signaling pathway
CD31	Platelet/endothelial cell adhesion molecule	20	MAPK, CD31 signaling pathway
FGFR2	Fibroblast growth factor receptor 2	17	MAPK, PI3K-Akt, RANKL signaling pathway
HIF-1	Hypoxia-inducible factor 1	15	HIF-1, PI3K-Akt signaling pathway

**TABLE 4:** The network of TFDR consisted of 18 candidate points.

No	Candidate	Molecular formula	2D conformer
1	Eriodictyol	C15H12O6	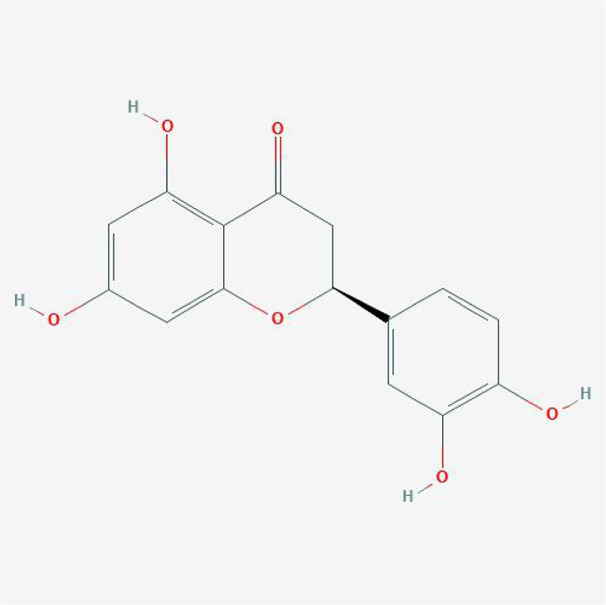
2	Digallate	C14H9O9-	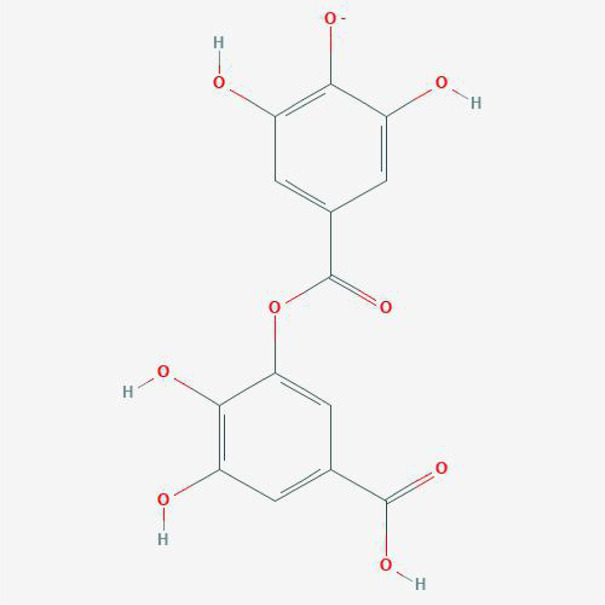
3	Luteolin	C15H10O6	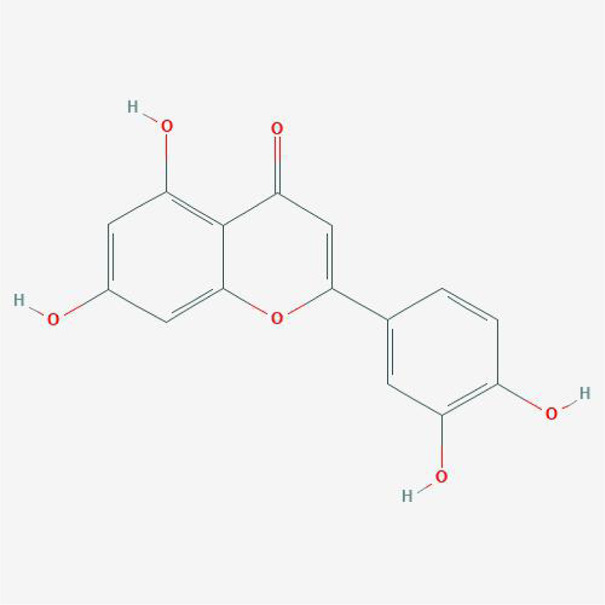
4	22-Stigmasten-3-one	C29H48O	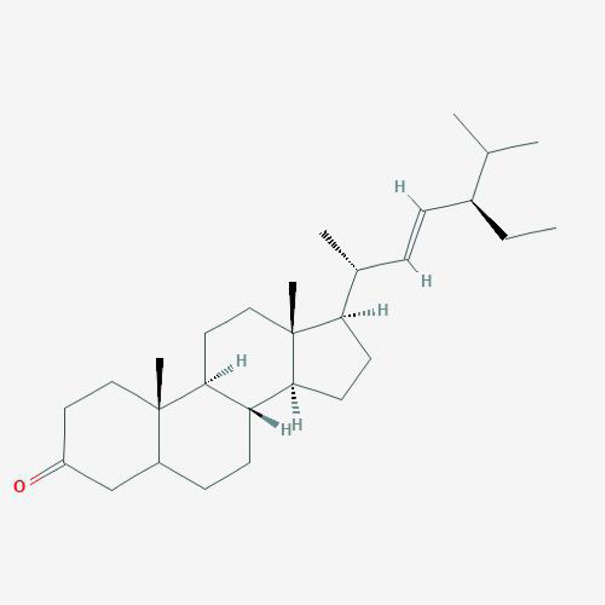
5	Cyclolaudenol acetate	C33H54O2	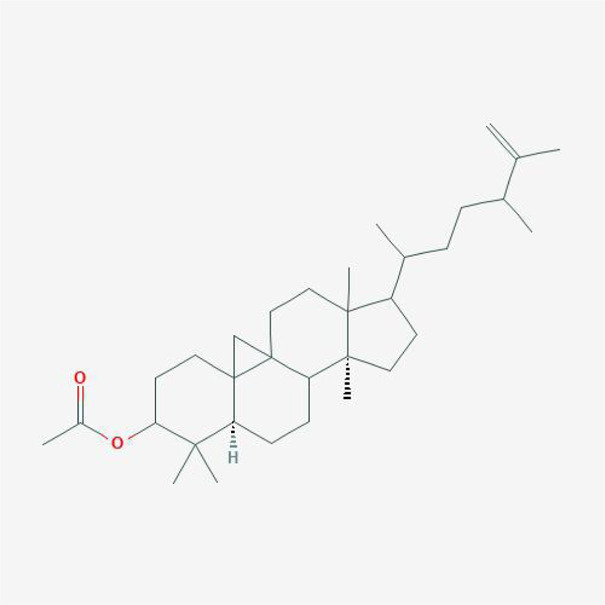
6	Cycloartenone	C30H48O	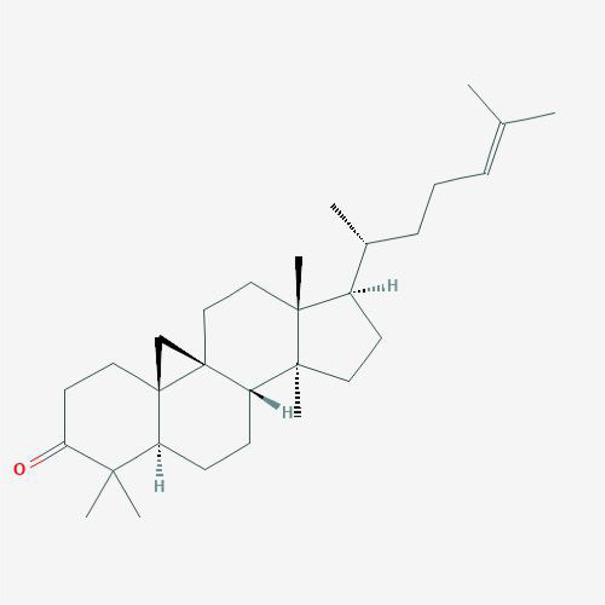
7	Cyclolaudenol	C31H52O	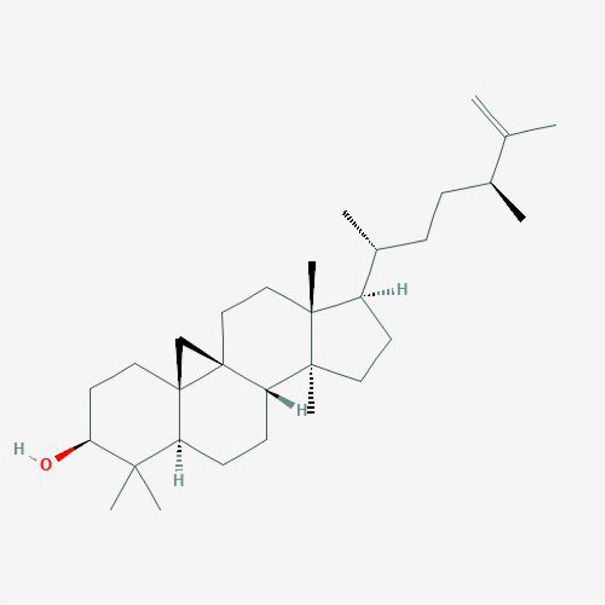
8	Davallioside A_qt	C25H29NO12	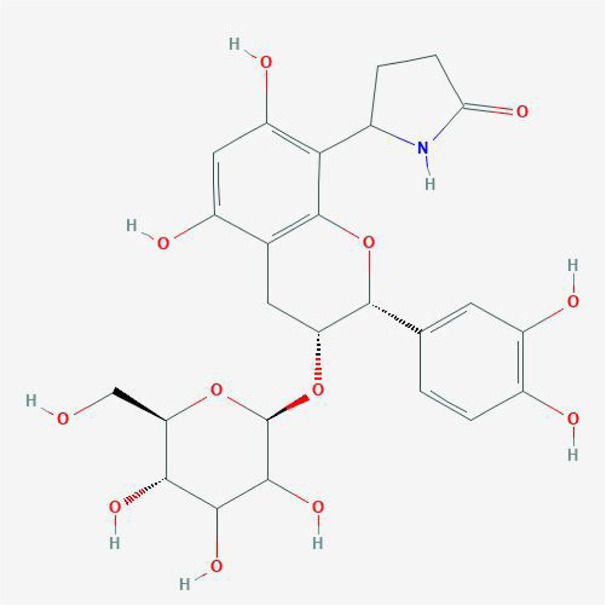
9	Marioside_qt	C22H34O10	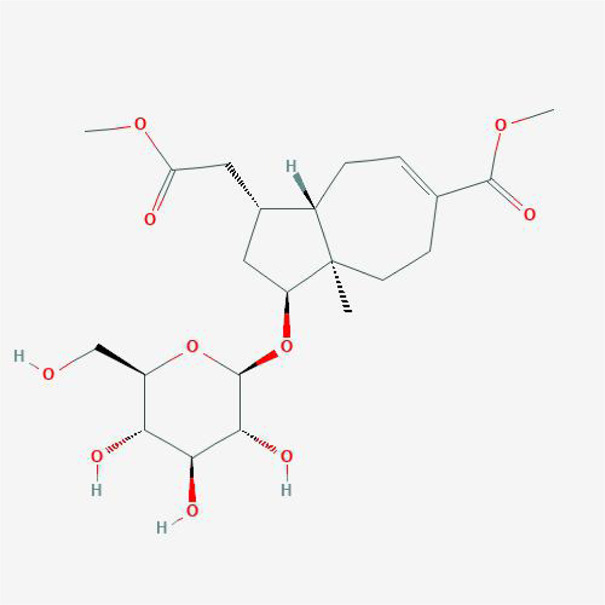
10	Xanthogalenol	C21H22O5	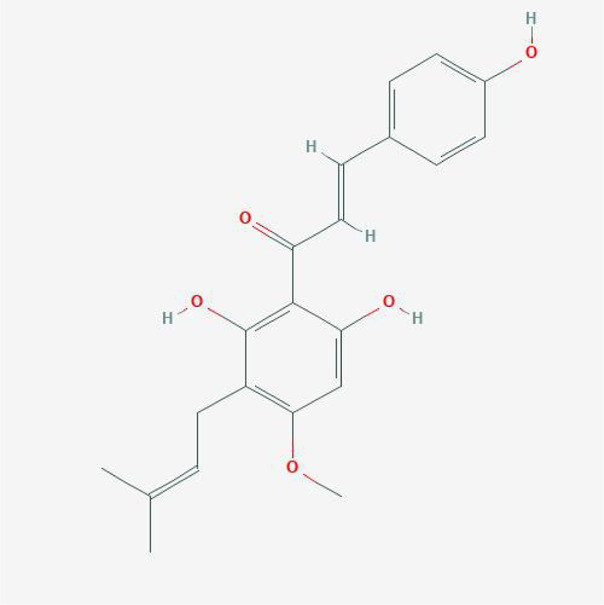
11	(2R)-5,7-Dihydroxy-2-(4-hydroxyphenyl)chromen-4-one	C15H12O5	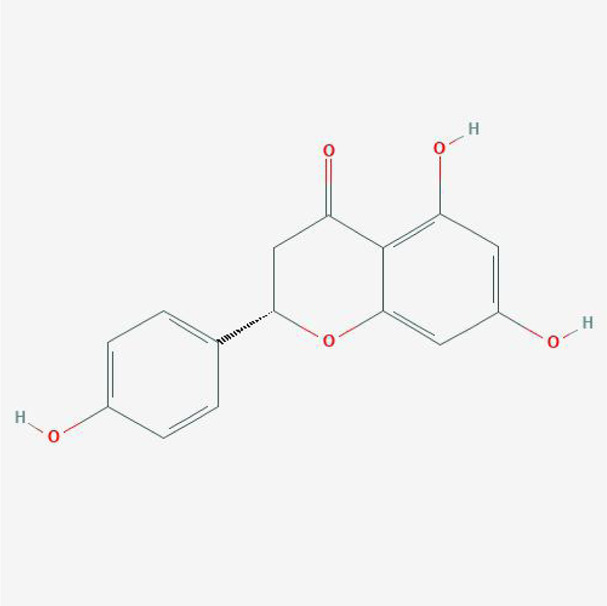
12	Aureusidin	C15H10O6	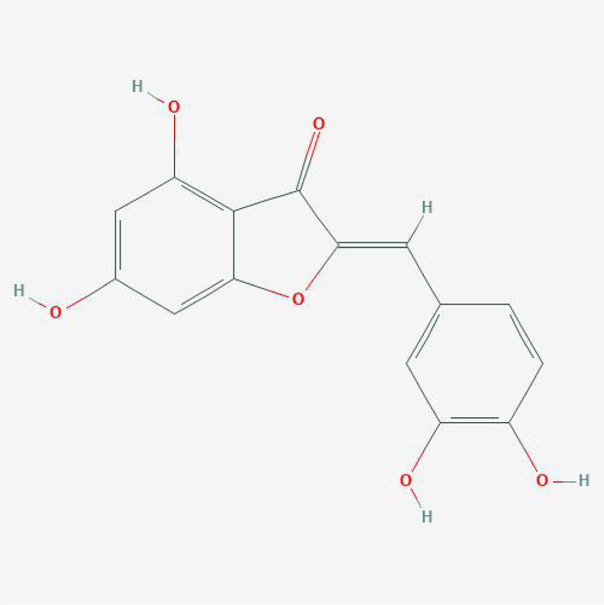
13	Eriodyctiol (flavanone)	C15H12O6	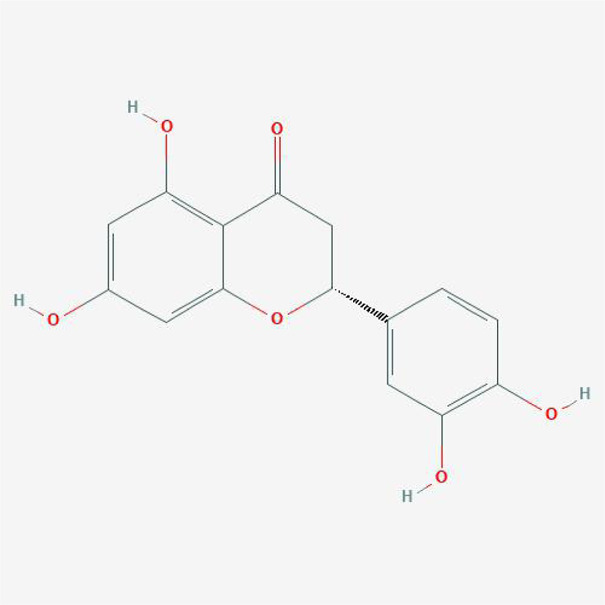
14	Stigmasterol	C29H48O	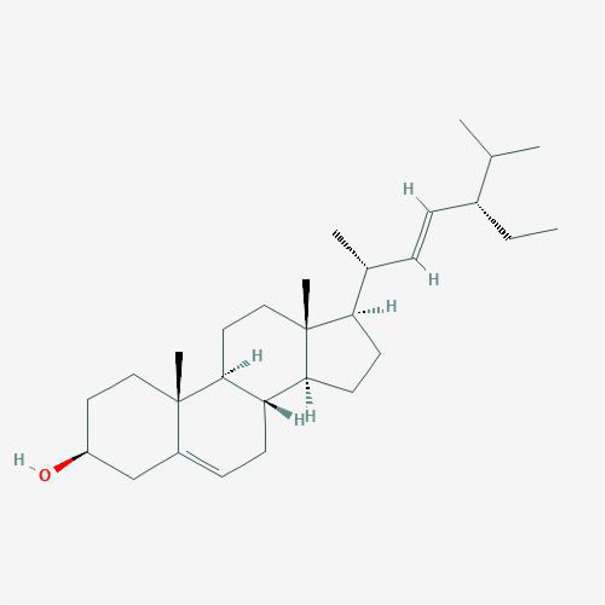
15	Beta-sitosterol	C29H50O	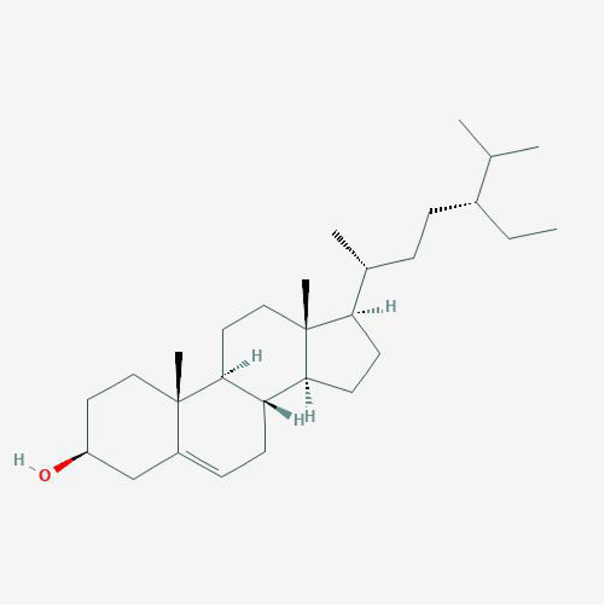
16	Kaempferol	C15H10O6	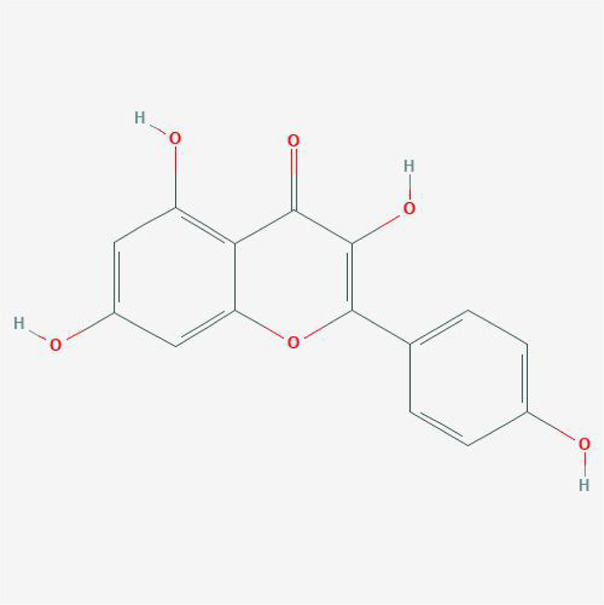
17	Naringenin	C15H12O5	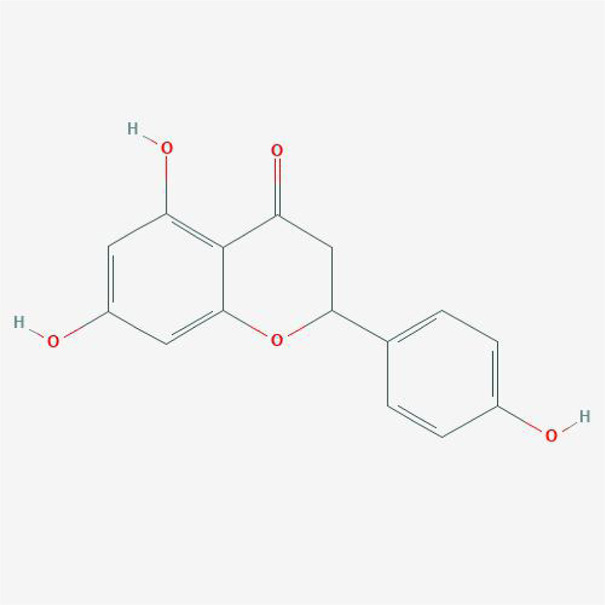
18	(+)−catechin	C15H14O6	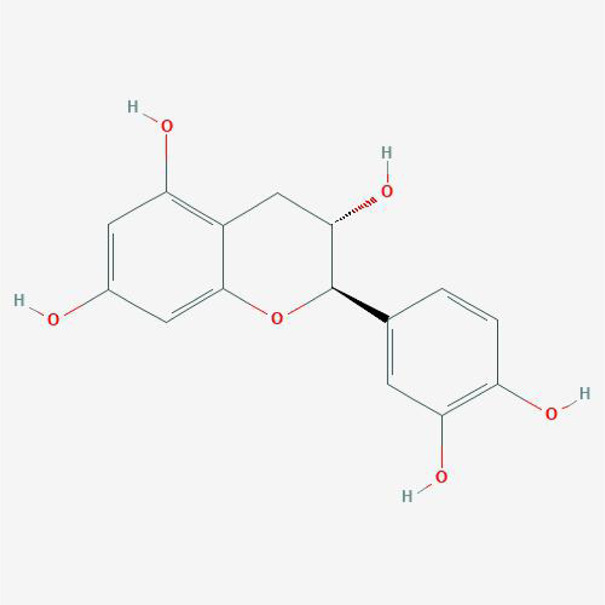

What’s more, this network showed the complex relationship between candidates and therapeutic targets, which contributed to identifying the curative features of TFDR in promoting osteogenesis (different components might act on the same targets meanwhile a targets could be regulated by multiple components).

### Biological Function Annotation and Pathway Enrichment Analysis

**FIGURE 2:**
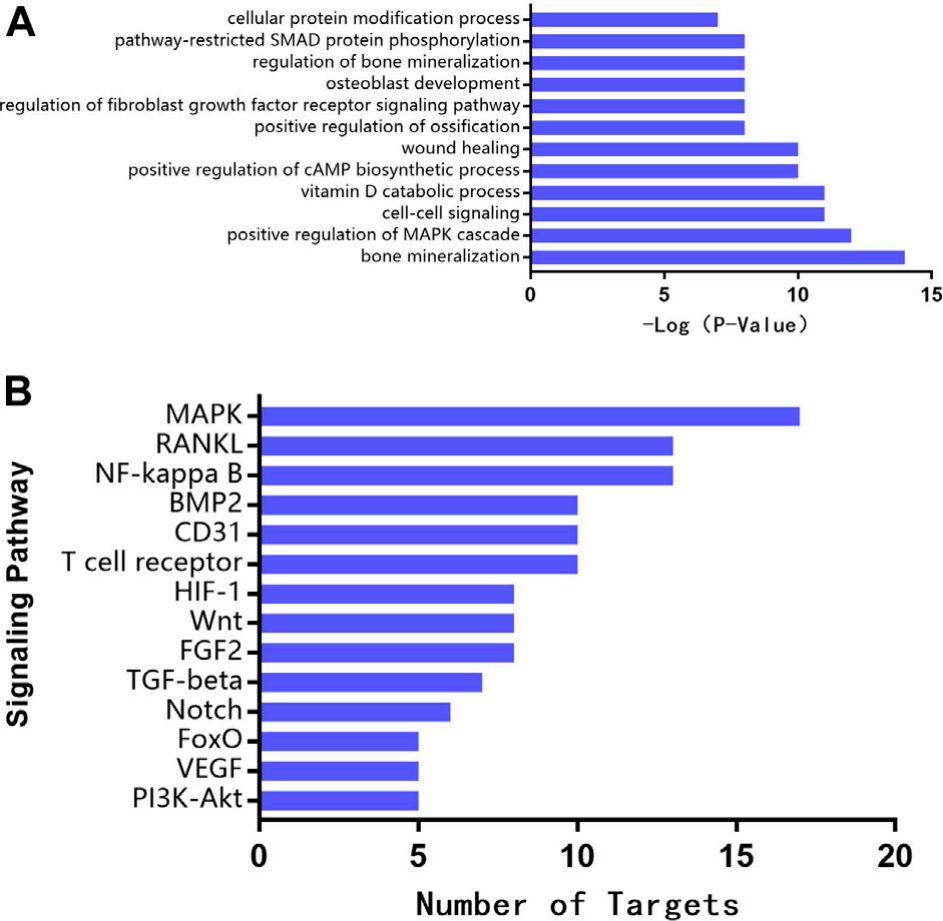
**(A)** Biological function annotation **(B)** Pathway enrichment analysis for therapeutic targets of TFRD.

In order to uncover the mechanism of TFDR in promoting osteogenesis and improving LBDs, we implemented function and pathway enrichment analysis for the potential therapeutic targets. As highlighted in Figure 2A, biological processes of these targets were mainly involved in bone mineralization, positive regulation of MAPK cascade, cell-cell signaling, etc., Moreover, the signaling pathway analysis data illustrated that MAPK, RANKL, NF-kappa B signaling pathway and so on were obviously enriched. Among the three signaling pathways, MAPK has the highest correlation with bone defects (Figure 2B). Meanwhile, we preliminarily speculated MAPK signaling pathway were more important in regulating osteogenesis because of more targets involving in it, in which p38 MAPK played the most important role(Zhang et al., 2018). And several previous studies have found the proliferation and osteogenic differentiation of BMSCs are inhibited by suppressing the p38 MAPK signaling pathway(Miele et al., 2000). Based on the pathway enrichment analysis, we hypothesized that TFDR improved LBDs by regulating the p38 MAPK signaling pathway. *In vivo* and *in vitro* experiments were conducted to elaborate the underlying regulating mechanism.

### Evaluating the Bone Reconstruction Level by X-Ray Scores

**FIGURE 3:**
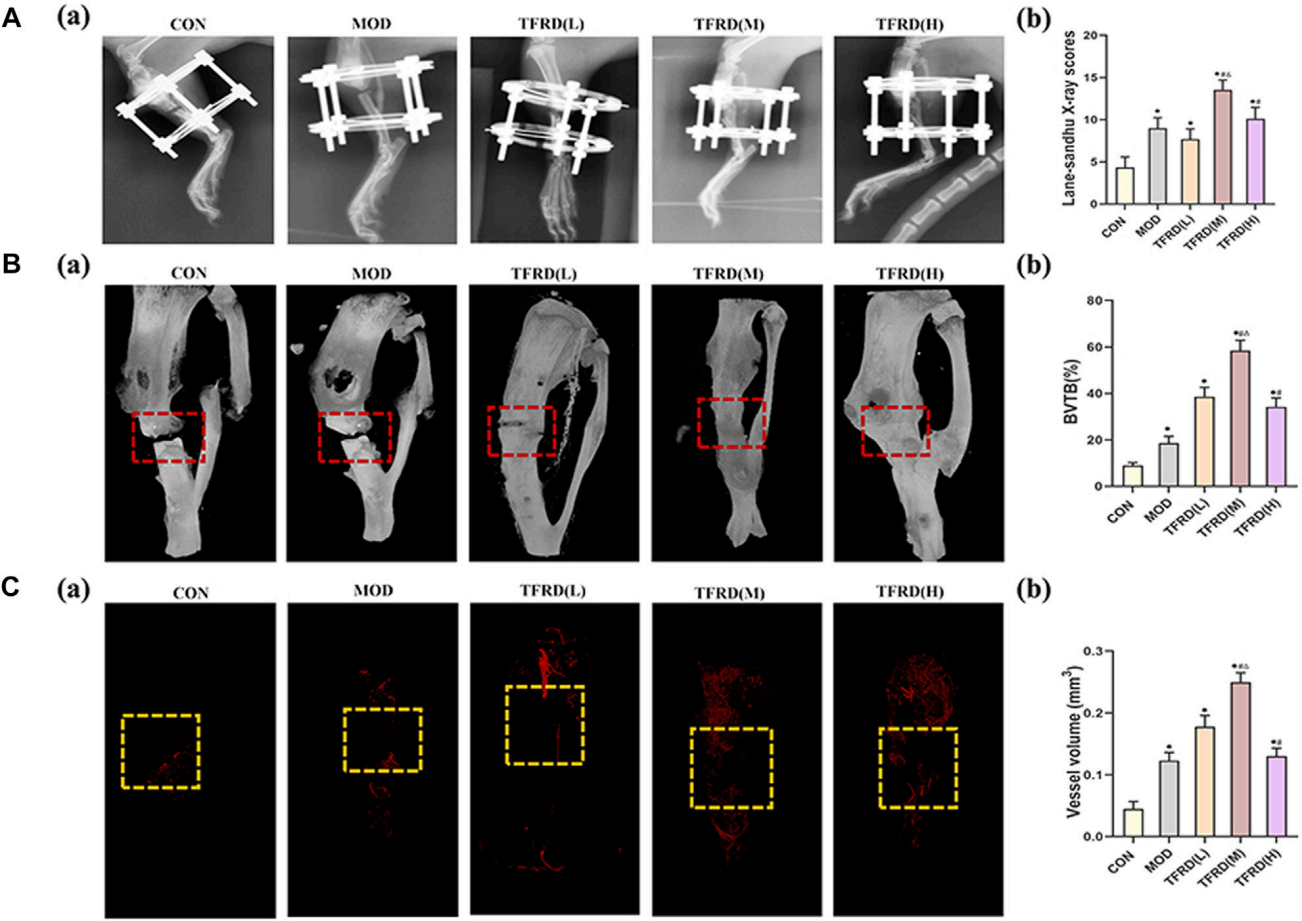
Evaluation of radiological, micro-CT images, angiogenesis of tibial bone repair of five groups **(A)**. Radiological evaluation of bone repair **(A)** (a). Representative radiographs of bone repair of the five groups at 12 weeks after surgery (*n* = 3 per group); **(A)** (b). Quantitative analysis of radiographic scores **(B)**. Representative micro-CT images of bone repair **(B)** (a). Three-dimensional reconstructed images of bone defects at 12 weeks after surgical dotted boxes indicate region of interest (ROI), representing bone distracted gaps (*n* = 3 per group); **(B)** (b). Quantification of bone tissue volume/total tissue volume and (BV/TV) insides bone distracted regions **(C)**. Evaluation of angiogenesis within the distracted gaps at 4 weeks after surgery **(C)** (a). Representative angiographs of the distracted gaps in the five groups (*n* = 3 per group) **(C)** (b). Quantification of vessel volume within the distracted regions (yellow dotted boxes indicate region of interest (ROI), representing bone distracted gaps). The data are expressed as the mean ± SEM of three independent experiments. *p < 0.05, compared with the control group; ^#^ *p* < 0.05, compared with the model group; ^△^ *p* < 0.05, compared the difference of the medium dose with the low and high doses in the TFDR group.

At the 12th week after surgery, the fracture line became more indistinct and more callus had jointed the two fracture ends in the bone defect area of rats in the TFDR groups and Model (MOD) group compared with the model group. Among the TFDR groups, TFDR medium dosage group had the densest callus, the fracture line was the vaguest, and the osteotomy gap nearly disappeared. All the radiological scores from X-ray films were showed in Figure 3A. By comparing the radiological scores in each group, it was significantly higher in the TFDR groups and MOD group than the control group. Moreover, X-ray scores were higher in the TFDR medium dosage group [TFDR (M) group] and TFDR low dosage group [TFDR (L) group] compared with the MOD group, suggesting that TFDR significantly promoted bone reconstruction in large tibial defect.

### Evaluating the Bone Repair Level by Micro-CT Scanning

The callus volume of rats in the TFDR groups were higher than that in the model group as illustrated by the Micro-CT scanning (Figure 3B). According to the results of quantitative analysis (Figure 3B), the BV/TV ratio of rats was higher in the TFDR groups and MOD group than that in the control group, but was lower in the MOD group than those in the TFDR group (all *p* < 0.05). The results above indicated that TFDR showing certain advantages in the osteogenesis coupling.

### The Positive Effect of TFDR on Vascular Network

The rats were perfused with Microfil to observed vascular formation in tibial defects at 4 weeks (Figure 3C). Sparse vascular network was observed in MOD group while more vessels were detected in TFDR group. Notably, the richest vascular network was observed in TFDR (M) group. For quantitative analysis, vessel volume was increased in TFDR groups and MOD group compared with CON group (*p* < 0.05). Moreover, vessel volume was increased in the TFDR (M) group and TFDR (L) group compared with the MOD group. And Lower vessel separation was observed in the TFDR (M) group, which suggested that TFDR in the medium dosage significantly improved vascular formation in large tibial defect.

### Expression of p38 MAPK Pathway-Related Genes

**FIGURE 4:**
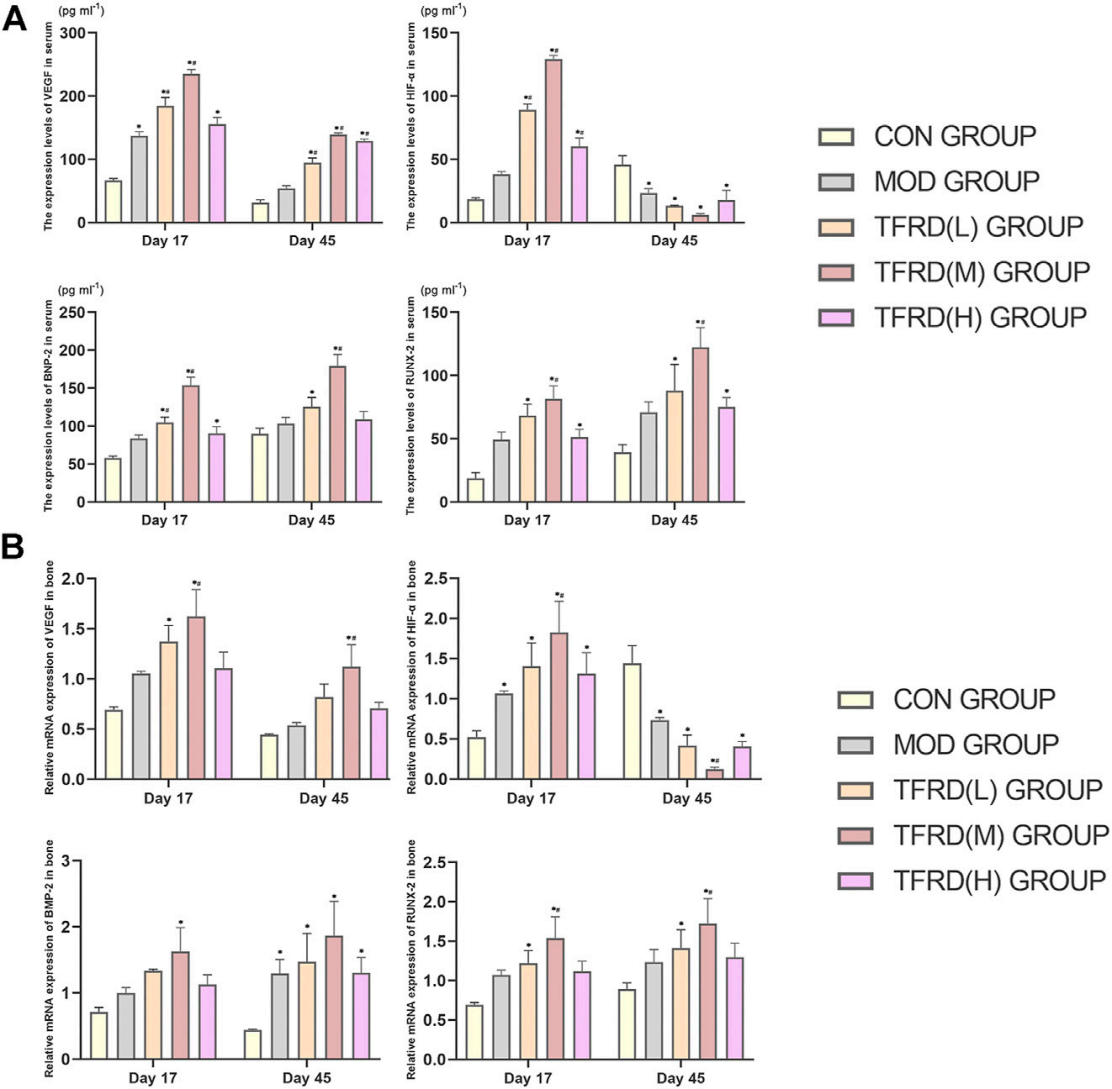
The expression levels of VEGF, HIF-1α, RUNX-2, and BMP-2 in serum and in bone respectively **(A)**. VEGF, HIF-1α, RUNX-2, and BMP-2 concentrations in serum of DO groups detected by ELISA (*n* = 3 per group); **(B)**. VEGF, HIF-1α, RUNX-2, and BMP-2 relative mRNA expression of DO groups detected by Real-Time PCR (*n* = 3 per group). The data are expressed as the mean ± SEM of three independent experiments. *p < 0.05, compared with the control group; ^#^ *p* < 0.05, compared with the model group.

In accordance with the outcomes of molecular studies (Figure 4), compared with the model group, the levels of VEGF, HIF-1α, RUNX-2 and BMP-2 expression in serum in the high, medium and low dosage TFDR groups were significantly increased (p < 0.05). What’s more, the relative mRNA expression levels in the high, medium and low dosage TFDR groups also were significantly increased when drawn a comparison with the model group. In addition, the medium doses of TFDR was more effective in enhancing the expression levels of VEGF, HIF-1α, RUNX-2 and BMP-2 and their relative mRNA.

### Evaluating the Morphological Structure by Histological Analysis

**FIGURE 5:**
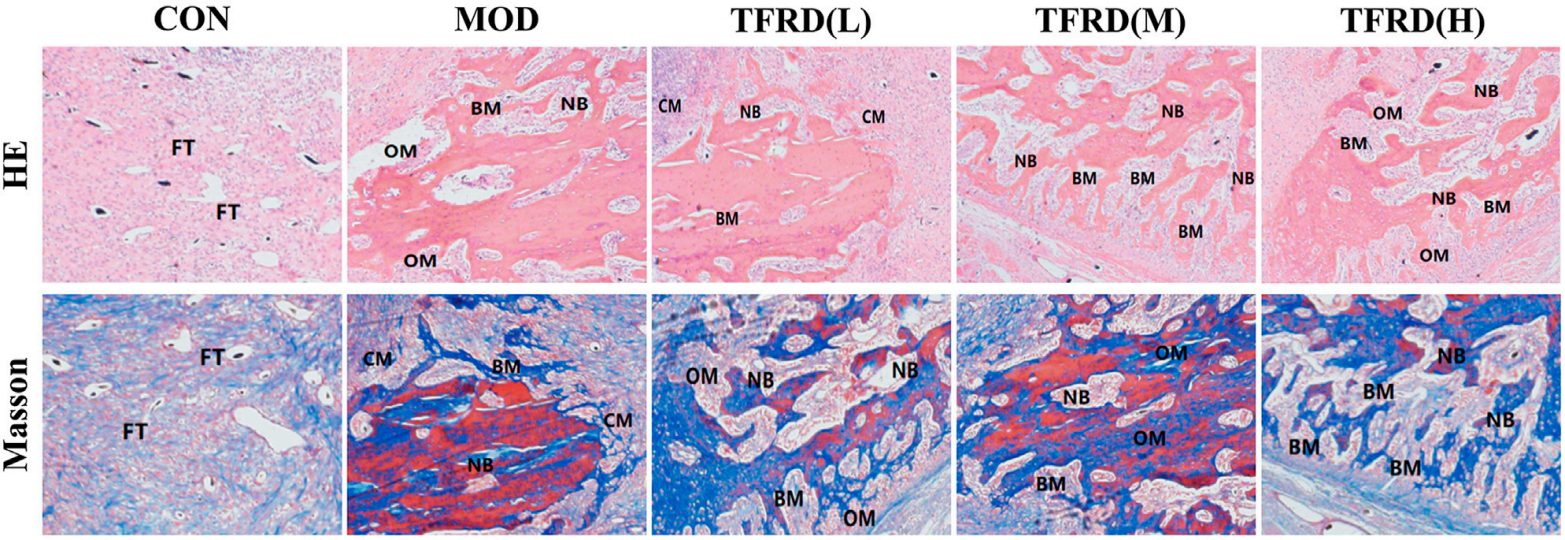
Histological analysis of newly formed tissues within distracted gaps at 12 weeks after surgery. Representative histological images of newly formed tissues within distracted gaps at 12 weeks after surgery (five random visual fields per section, three sections per staining, nine sections per rat). From up to down: H&E, Masson’s. We mainly observed six points from the histomorphology assay, including newly formed bone (NB), bone marrow (BM), osteoid matrix (OM), chondroid matrix (CM), and fibrous tissue (FT), to evaluated the bone reconstruction level.

Histological evaluation showed that the newly formed bone (NB**)**, bone marrow (BM), osteoid matrix (OM), chondroid matrix (CM) and the number of osteoblasts in the newly formed bone tissue of the rats were significantly increased in TFDR groups (Figure 5). While the less newly formed bone tissue was observed in the MOD group and the fibrous tissue (FT) was only seen in the CON group. Interestingly, NB and BM were significantly clustered in TFDR (M) group, while OM were mainly observed in the TFDR (L) groups and TFDR (H) groups, which implied that TFDR promote differentiation and capacity of mineralization of bone marrow stromal cells and contribute to the prevention and treatment of bone defect.

### BMSCs Morphology Identification by Flow Cytometry

**FIGURE 6:**
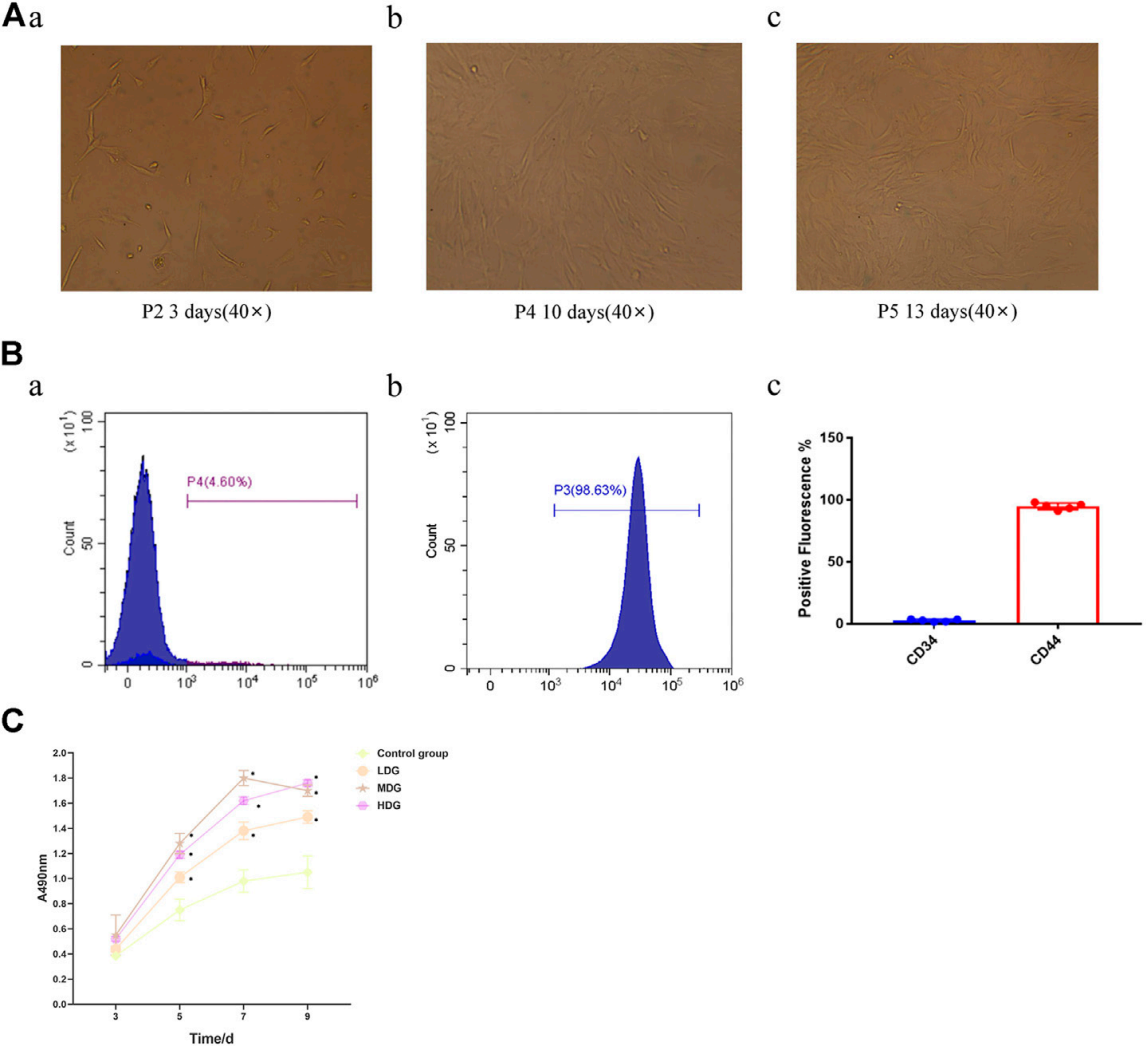
Identification and cell viability assay of BMSCs **(A)** photomicrograph of BMSCs morphology from Sprague-Dawley rats (40 ×) (a)The BMSCs morphology was extended to fusiform or polygonal after incubation for 3 days; (b) The BMSCs morphology was merged with each other after incubation for 10 days (c) The BMSCs morphology was stretched out like a uniform shape of spindle by degrees after incubation for 13 days. **(B)** Surface marker expression of BMSCs (a)The expression of surface marker CD44 indicated negative results; (b) The expression of surface marker CD34 showed negative results; (c) Quantification of surface marker expression of BMSCs. **(C)** The CCK-8 assay of BMSCs exposed to 3, 5, 7, and 9 days (*p＜0.05 vs. control group). The data are expressed as the mean ± SEM of three independent experiments.

The BMSCs, just planted on the culture plate, were in shape of round mixed with other cells. After culturing for 24 h, the BMSCs grew adherent to the wall with small quantity and the spindle shape. 3 days later, the adherent BMSCs showed an increase both in size and quantity, which extended gradually into fusiform or polygonal, and began to grow in clusters. After incubation for 7–9 days, the number of colonies was gradually increased and merged with each other. When it came to the 9th–11th days, the cell fusion state could be achieved more than 80%. The cells were in round shape after passage and adherent to the wall within 12 h of inoculation. The BMSCs morphology was stretched out like a uniform shape of spindle by degrees. The cell proliferation rate grew quickly that the bottom of the bottle was covered with over 80% cells in shape of vortex on day 5 of culture. The BMSCs morphology on the 3rd, 10th, and 13th day of culture are displayed in Figure 6A.

### Expression of Surface Markers of BMSCs

The surface marker CD44 accounted for 4.60%, indicating negative results (Figure 6B a), while the expression rate of surface marker CD34 of the third-generation BMSCs was 98.63%, indicating positive results (Figure 6B b).

### CCK-8 Assay

The absorbance value at each time point was higher in the TFDR treatment groups compared with the control group, and the difference was statistically significant (*p* < 0.05). The TFDR medium dosage group and high dosage group showed higher absorbance value at each time point than the TFDR low dosage group. On the 7th and 9th day, there was no significant difference between the TFDR medium dosage group and high dosage group. The TFDR groups produced beneficial effects on BMSCs proliferation after 3–5 days of culture, while entered a platform period during 7th to 9th days (Figure 6C).

### ALP Staining and Activity Detection

**FIGURE 7:**
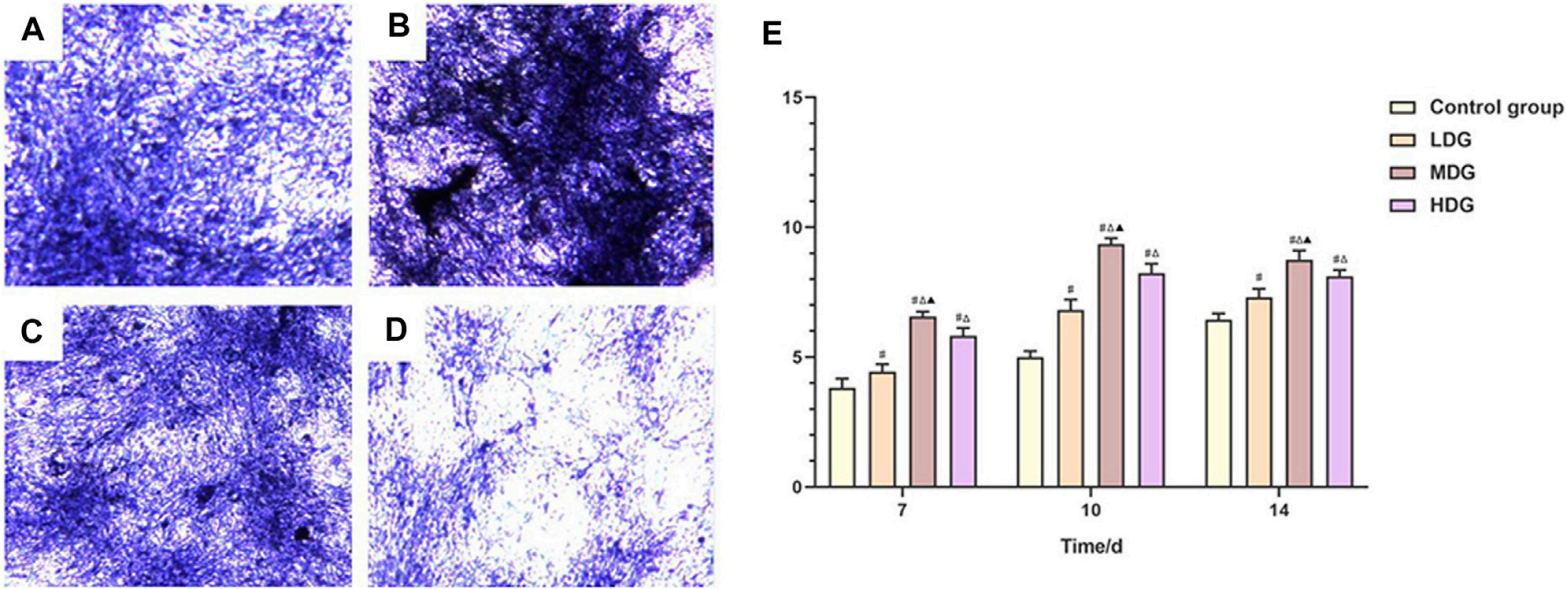
The ALP staining assay is performed to evaluate ALP activity after incubated for 10 days **(A)**. TFDR low dosage group; **(B)**. TFDR medium dosage group; **(C)**. TFDR high dosage group; control group; **(D)**. control group **(E)**. The ALP activity at each time point of the control group, TFDR low dosage group, TFDR medium dosage group, and TFDR high dosage group. The data are expressed as the mean ± SEM of three independent experiments. ^#^ *p* < 0.05 vs. control group, ^∆^ *p* < 0.05 vs. TFDR low dosage group, ^▲^ *p* < 0.05 vs. TFDR high dosage group.

When BMSCs were cultured in corresponding culture medium for 7–14 days, the ALP activity of the control group, TFDR low dosage group and TFDR medium dosage group was gradually increased over time and maintained at a high level. The ALP activity of TFDR medium dosage group reached a relative high level after culture for 10 days, while there was a fallen tendency with time increased. The ALP activity of each group on day 10 of culture is shown in Figures 7A–D. The TFDR treatment groups showed higher ALP activity at each time point in comparison with the control group (*p* < 0.05). The ALP activity of TFDR medium dosage group at each point was higher than that of TFDR low dosage group and TFDR high dosage group (*p* < 0.05). The ALP activity at each time point of each group is summarized in Figure 7E.

### Quantity Evaluation of Mineralized Nodules

**FIGURE 8:**
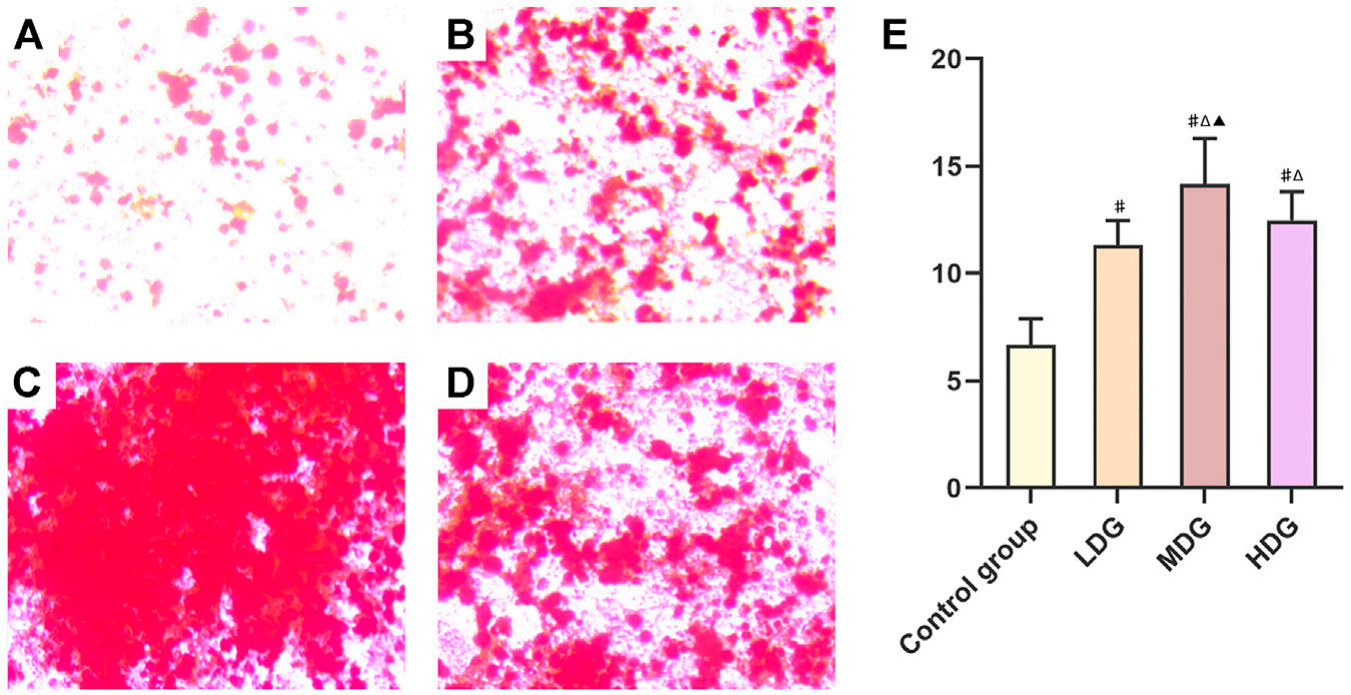
Representative images of BMSCs with the alizarin red staining to determine the mineralized nodules. **(A)** Control group; **(B)** TFDR low dosage group; **(C)** TFDR medium dosage group; **(D)** TFDR high dosage group; (E) The mineralized nodules at each time point of the control group, TFDR low dosage group, TFDR medium dosage group, and TFDR high dosage group were evaluated. The data are expressed as the mean ± SEM of three independent experiments. #*p* < 0.05 vs. control group, ^∆^ *p* < 0.05 vs. TFDR low dosage group, ^▲^ *p* < 0.05 vs. TFDR high dosage group.

After 21 days of intervention, mineralized nodules were formed in each group. The alizarin red staining showed red dense nodules with uneven size in each group (Figures 8A–D). Compared with the control group, the number of mineralized nodules in TFDR intervention groups increased significantly (*p* < 0.05). The mineralized nodules in TFDR medium dosage group accounted for the largest quantity in comparison with TFDR low and high dosage group (*p* < 0.05). The number of mineralized nodules in each group is shown in Figure 8E.

### Measurement of BMP-2, p38 MAPK, VEGF, RUNX-2, and HIF-1αmRNA Levels by qRT-PCR

**FIGURE 9:**
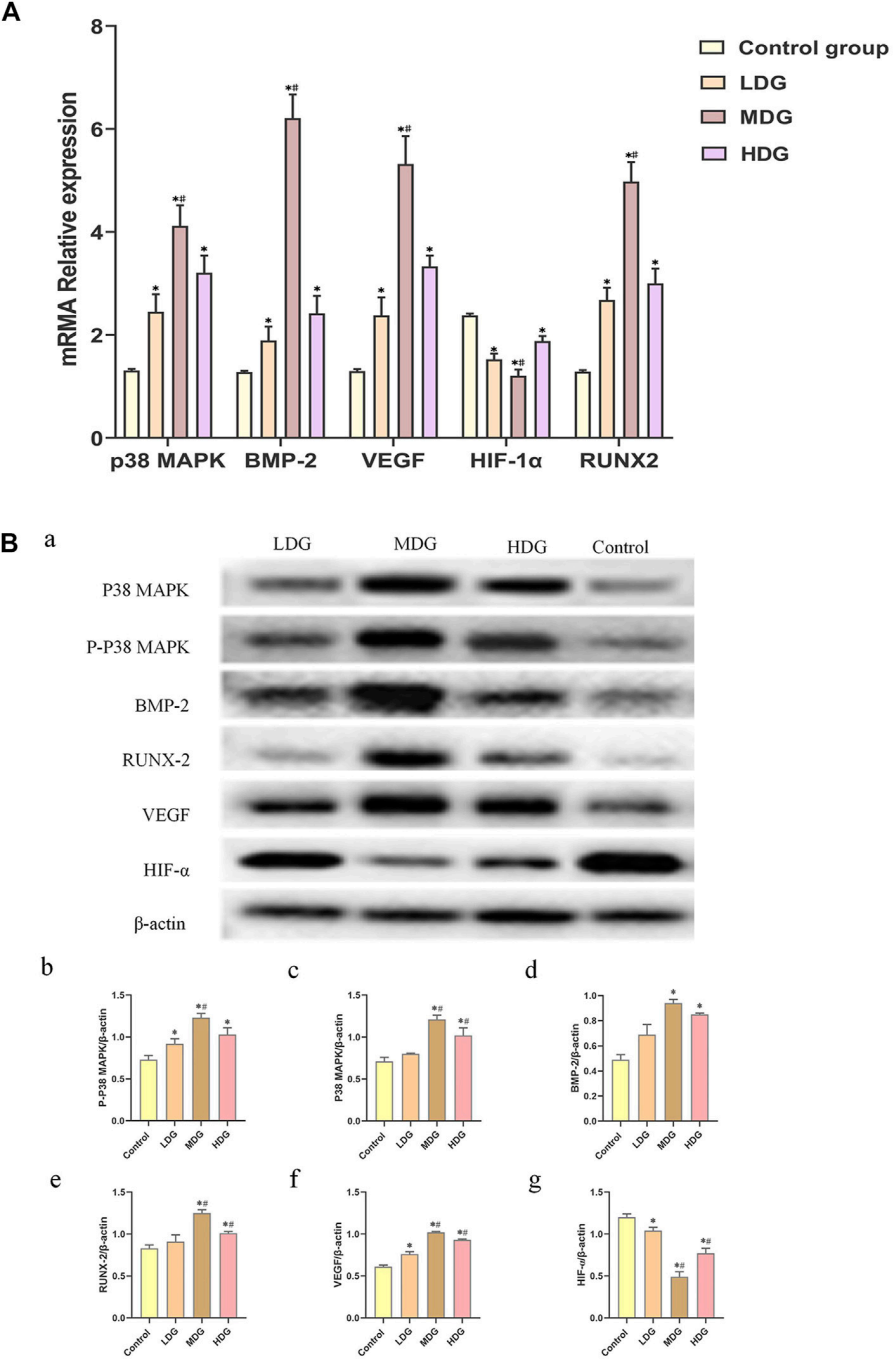
The expressions of p38 MAPK, BMP-2, VEGF, HIF-1α, and RUNX-2 mRNA on BMSCs by quantitative real-time PCR. The data are expressed as the mean ± SEM of three independent experiments. *p < 0.05 vs. control group, ^#^ *p* < 0.05 vs. the TFDR low dosage group, TFDR high dosage group. **(B)** (a) p38 MAPK, p-p38 MAPK, BMP-2, RUNX-2, VEGF, and HIF-1α protein expression on BMSCs detected by western blot analysis. **(B–G)** were statistical analysis of **(A)**. The data are expressed as the mean ± SEM of three independent experiments. *p < 0.01 vs. the Control group; *p < 0.01 vs. the TFDR low dosage group.

After intervention with TFDR-containing serum for 7 days, the expression of BMP-2, p38 MAPK, VEGF, RUNX-2 mRNA levels in the TFDR treatment group were markedly increased compared with the control group (*p* < 0.05), the expression of HIF-1αmRNA in the TFDR treatment group was significantly lower than that in the control group (*p* < 0.05). The TFDR medium dosage group showed a significant increased expression of BMP-2, p38 MAPK, VEGF, RUNX-2, and HIF-1α mRNA compared with the control group, TFDR low dosage group and TFDR high dosage group (*p* < 0.05) (Figure 9A).

### Measurement of BMP-2, p38 MAPK, p-p38 MAPK, VEGF, RUNX-2, and HIF-1α Protein Expression by Western Blot Analysis

The results of western blot analysis showed an enhanced phosphorylation of p-p38 MAPK level in BMSCs, which reflected the activity of p38 MAPK. TFDR treatments led to an obvious increase in the protein expression of BMP-2, p38 MAPK, p-p38 MAPK, VEGF and RUNX-2 compared with the control group. What’s more, the expression of BMP-2, p38 MAPK, p-p38 MAPK, VEGF and RUNX-2 protein in the TFDR medium dosage group were significantly higher than those in the control group, TFDR low dosage group and TFDR high dosage group (Figure 9B).

## Discussion

LBDs refers to the length of a long bone defect formed by various reasons that exceeds 1.5 times of the long bone or the bone defect is larger than 1/5–1/4 of the bone(Schlegel et al., 2006). There are many ways to treat LBDs, including Masquelet technique, autologous bone transplantation, allogeneic bone transplantation, xenograft bone transplantation, artificial bone transplantation, and the DO technique created by Ilizarov.

At present, DO has become one of the indispensable methods for various bone diseases in orthopedics field. It has greatly improved the quality of life of millions of people around the world and had a profound impact on the treatment of orthopedic diseases(Gubin et al., 2013). Inspired by a vertical bow harness on a carriage, Dr. Ilizarov observed the phenomenon of DO in which traction can cause callus formation, and named it the *Law of Tension-stress* (LTS) (Ilizarov, 1989). DO stimulates the growing of bone and soft tissue through controlling and mechanically applying tensile stress, so that the bone and soft tissue can grow with the stress direction. In addition, researchers have applied DO with external fixation to treat 19 cases of tibia segmental bone defects. Results showed that bone defects were healed in all cases, the length of the affected limb was 2 cm less than the normal side, and the fracture was healed completely(He, 2015). Using the principle of DO to treat LBDs especially when the defect range is over 5 cm is of benefit(Vukasinovic et al., 2012; Long et al., 2013). However, after the completion of bone transport, it takes 6 months or longer for the new bone tissues, which formed by the closed bone block and the lengthened osteotomy zone, to heal and become mineralization. Therefore, it’s necessary to find a way to accelerate the bone formation, shorten the course of disease and reduce the occurrence of complications(Long et al., 2013; He, 2015).

TCM has thousand years of history in treating fractures and has proven effective in practice. Hulth pointed out that bone lengthening is actually a continuous regeneration of bone healing, that is, the healing mechanism of large bone after DO surgery is the same as fracture healing(Hulth, 1989). Studies showed that Chinese medicine was effective at different stages of fracture healing. Zhang Li et al. proved that in the early stage of fracture, Chinese medicine promoted VEGF expression and vascular regeneration and reconstruction. The peak of VEGF expression was about 1 week, and then began to decline(Zhang and Ye, 2004). Wen-ling Wang, et al. showed that Chinese medicine significantly increased the number of osteoblasts, intracellular ALP levels, and nodule numbers, while inhibited the osteoclast activity. In addition, 1000 μg/ml of Danggui Buxue Decoction was able to stimulate p-ERK and p-JNK signaling pathway, which is highly promising for accelerating fracture healing in the middle or late healing periods(Wang et al., 2014). By using rabbits’ radius bone defect models and treating them in different stages with Chinese medicine, a study observed the VEGF expression in various tissues of the callus was better than that in the non-stage treatment group(Xu et al., 2010). Furthermore, experiment studies showed the promoting the VEGF expression, BMP-2, VEGFm RNA, and BMP-2m RNA in fracture healing in rabbits by Chinese medicine featured by reinforcing tendon and bone was better than other treatment groups in outer periosteum, inner periosteum and bone marrow cavity (Bi, 2013), whose results were quite similar to ours.

The total flavonoid in the DR accounted for 1.42% and the content of naringin was 1%. The active ingredient of DR was TFDR, which is characterized with promoting blood circulation, removing blood stasis, repairing bones and enhancing the function of myocardial cells (Fang et al., 2006). Contemporary pharmacological researches showed that TFDR has the effects of preventing osteoporosis, lowering blood lipids, promoting fracture healing. What’s more, TFDR could promote the bone calcium absorption, increase the biological level of blood phosphorus and blood calcium, contributing to the formation of bone salt deposition and bone calcification. In the process of fracture healing, TFDR is able to increase the thickness of the callus, improve the quality of fracture healing and raise the expression of mRNA, BMP-2m RNA, and TGF-β1 genes(Lee et al., 2014). The past animal experiments concluded that TGF-β1 of the callus tissue in the TFDR group was significantly higher than that of the control group in immunohistochemical staining. Researchers normally speculated that the mechanism of TFDR in healing fractures may be: 1) promoting the expression of TGF-β1 in the callus tissue; 2) increasing the concentration of blood phosphorus and calcium; 3) increasing the activity of ALP in serum(Yang et al., 2013).

In this study, based on network pharmacology, we found that the MAPK is the most related signaling pathway in bone defects. The MAPK signaling pathway mainly consists of ERK1/2 signaling pathway, p38MAPK signaling pathway and JNK signaling pathway. However, the p38 signaling pathway is an important part of the MAPK signaling pathway. Through external stimuli such as cytokines, extracellular signals are transduced into cells to exert biological regulation effects, which could improve body inflammation and regulate cell growth, differentiation and death as well(Ono and Han, 2000). The proliferation and differentiation of BMSCs is regulated by the p38 MAPK signaling pathway, which cause a series of changes in ALP, Runx2 and other osteogenic related genes(Lee et al., 2002). Another study found that low intensity and high frequency vibration could induce osteogenic differentiation of BMSCs and high expression of Runx2 and ALP through the p38 MAPK signaling pathway(Lu et al., 2018). Several studies have found that the proliferation and differentiation of BMSCs were inhibited by inhibiting the p38 MAPK signaling pathway. What’s more, with the activation of p38 MAPK signaling pathway, the expression of BMP-2, p38 MAPK, p-p38 MAPK, VEGF, RUNX-2, and HIF-1α and their relative mRNA enhanced in the *in vitro* experiment of this study, thus verifying that p38 MAPK plays a part in BMSCs osteogenesis rather than ERK1/2 or JNK MAPK pathway(Zhang et al., 2018; Jiang et al., 2019).

VEGF plays an important role in regulating the vascular growth. At present, VEGF is one the best growth factors in inducing angiogenesis in bone tissue engineering. VEGF has been recognized as a key factor in the formation of vessels and is highly specific for directly inducing the proliferation of vascular endothelial cells. What’s more, VEGF is able to make intravascular protein extravasate by increasing capillary permeability, and provide suitable conditions for the migration of vascular endothelial cells and the formation of capillary network (Henry, 1999).

In addition, through conditional knocking out the over expression HIF1α of VHL in osteoblasts, researchers found that the bone mass and VEGF expression in mice were increased significantly after birth, and could resist bone loss in older mice(Weng et al., 2014). This study indicated that in the process of bone formation and repair, the VEGF-dependent vessel growth is the key to the bone formation inside cartilage. Moreover, interaction between VEGF and BMP regulates the bone remodeling and vessel formation. Osteoblasts produce VEGF to stimulate endothelial cell proliferation, while endothelial cells express BMP2 to stimulate osteoblast progenitor cell differentiation(Busletta et al., 2011; Liu et al., 2012).

In this study, we found that the viability of BMSCs in the TFDR high dosage group started to decline from the 7th to the 9th day in CKK-8 assay. Moreover, Jeong., et al. showed that the cells cultured with 0.2 mg/ml of TFDR had the most vigorous growth by MTT. Although the above or below TFDR concentration of cells proliferated more vigorously than the standard group, the experimental results indicated that 0.2 mg/ml TFDR has the most optimal effect on promoting the proliferation of BMSCs, which is quite similar to the results of our study(Jeong et al., 2003). Another study showed that high concentration of TFDR has a harmful effect on BMSCs and most of them broke off the wall and floated to death in a short period of time. Moreover, these cells were hard to culture for a long time, and the residual cells also become non-morphological. The surface of these cells were rough and shrunken with some cells only debris remained, demonstrating that high concentration of TFDR affected the growth of BMSCs and caused cell death or apoptosis(Chen, 2005; Li et al., 2006). In addition, in the course of this experiment, we found that the two rats in the high-concentration TFDR group had mild gastrointestinal reactions. This may be one of the reasons why the experimental results of the high-concentration TFDR intervention group were not as ideal as the medium-concentration TFDR intervention group. Therefore, we will further study the association of TFDR dosage with bone formation and mineralization.

The limitation of study was that a positive control drug group in the animal experiment was not included. Bone morphogenetic protein and parathyroid hormone were currently effective and certified by the Food and Drug Administration. However, the effectiveness of these two drugs were not ideal as expected in clinical practice because they were unstable in efficacy. Therefore, we had not put these two drugs into our consideration in the animal experiment, which is also a limitation in the clinical treatment of LBDs.

There are many different therapeutic targets being tested for LBDs. However, most treatments highlight the role of one cell, one cytokine, or one signaling molecule, without considering the complexity of LBDs, thus cannot produce anticipated therapeutic effects. TFDR, as a new ingredient affecting p38 MAPK signaling pathway, up-regulating the expression of VEGF, HIF-1α, RUNX-2 and BMP-2, finally promoting the differentiation of BMSCs, showed a promising future, and we hope TFDR could be widely used for treating LBDs soon.

## Data Availability

The datasets presented in this study can be found in online repositories. The names of the repository/repositories and accession number(s) can be found in the article/Supplementary Material.
