# Corrigendum: Exploring the Mechanism of Total Flavonoids of Drynariae Rhizoma to Improve Large Bone Defects by Network Pharmacology and Experimental Assessment

**DOI:** 10.3389/fphar.2021.739503

**Published:** 2021-07-23

**Authors:** Weipeng Sun, Minying Li, Lei Xie, Zhexing Mai, Yan Zhang, Lieliang Luo, Zijian Yan, Zige Li, Hang Dong, Feng Huang, Zhen Shen, Ziwei Jiang

**Affiliations:** ^1^ The First School of Clinical Medicine, Guangzhou University of Chinese Medicine, Guangzhou, Guangdong Province, China; ^2^ Department of Orthopaedics, Kunming Municipal Hospital of Traditional Chinese Medicine, Kunming, Yunnan Province, China; ^3^ Department of Orthopaedics, The First Affiliated Hospital of Guangzhou University of Chinese Medicine, Guangzhou, Guangdong Province, China; ^4^ Medical College of Acupuncture-Moxibustion and Rehabilitation, Guangzhou University of Chinese Medicine, Guangzhou, Guangdong Province, China; ^5^ Science and Technology Innovation Center, Guangzhou University of Chinese Medicine, Guangzhou, Guangdong Province, China; ^6^ The Second School of Clinical Medicine, Guangzhou University of Chinese Medicine, Guangzhou, Guangdong Province, China

**Keywords:** drynariae rhizoma, experimental assessment, gusuibu, large bone defects, network pharmacology

In the original article, there was a mistake in the legend for **Figure 8** and Figure 9 as published. In **Figure 8**, the numbering of figure legends was incorrect. In Figure 9, the description of previous Figure 9 was not detailed enough. The correct legends appear below.

“**FIGURE 8** | Representative images of BMSCs with the alizarin red staining to determine the mineralized nodules. **(A)** Control group; **(B)** TFDR low dosage group; **(C)** TFDR medium dosage group; **(D)** TFDR high dosage group; **(E)** The mineralized nodules at each time point of the control group, TFDR low dosage group, TFDR medium dosage group, and TFDR high dosage group were evaluated. The data are expressed as the mean ± SEM of three independent experiments. ^#^ *p* < 0.05 vs. control group, ^Δ^ *p* < 0.05 vs. TFDR low dosage group, ^▲^ *p* < 0.05 vs. TFDR high dosage group.”

“FIGURE 9 | The expressions of p38 MAPK, BMP-2, VEGF, HIF-1α, and RUNX-2 mRNA on BMSCs by quantitative real-time PCR. The data are expressed as the mean ± SEM of three independent experiments. *p < 0.05 vs. control group, ^#^ *p* < 0.05 vs. the TFDR low dosage group, TFDR high dosage group. **(B)** (a) p38 MAPK, p-p38 MAPK, BMP-2, RUNX-2, VEGF, and HIF-1αprotein expression on BMSCs detected by western blot analysis. (b)–(g) were statistical analysis of (a). The data are expressed as the mean ± SEM of three independent experiments. *p < 0.01 vs. the Control group; ^#^ *p* < 0.01 vs. the TFDR low dosage group.”

In addition, there was a mistake in Figure 3, Figure 7 and Figure 9 as published. The authors uploaded the wrong version of Figure 3 and Figure 7, and uploaded the previous version of Figure 9 by mistake. The corrected Figure 3, Figure 7 and Figure 9 appear below.

The authors apologize for this error and state that this does not change the scientific conclusions of the article in any way. The original article has been updated.

**FIGURE 3:**
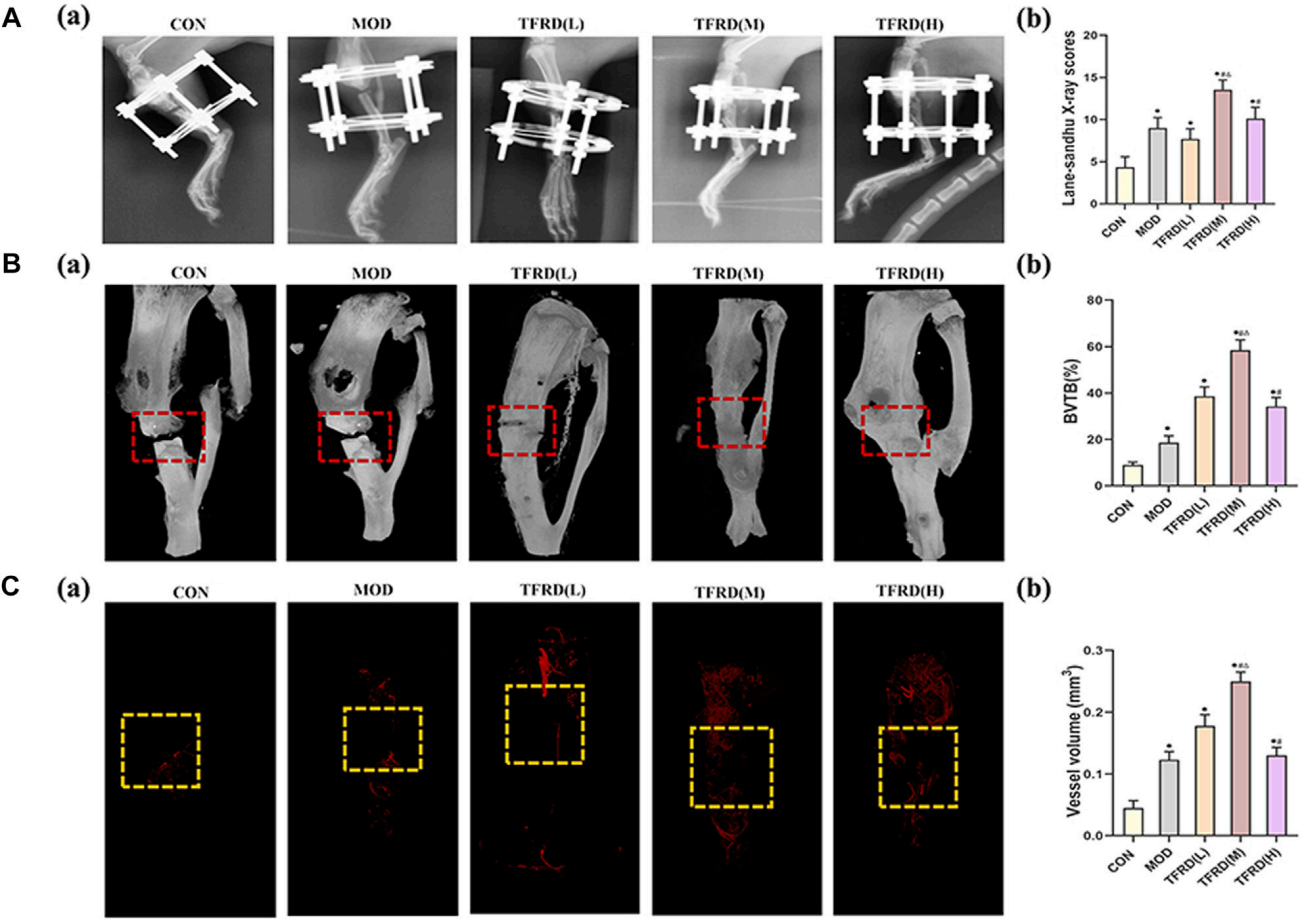
Evaluation of radiological, micro-CT images, angiogenesis of tibial bone repair of five groups **(A)**. Radiological evaluation of bone repair **(A)** (a). Representative radiographs of bone repair of the five groups at 12 weeks after surgery (*n* = 3 per group); **(A)** (b). Quantitative analysis of radiographic scores **(B)**. Representative micro-CT images of bone repair **(B)** (a). Three-dimensional reconstructed images of bone defects at 12 weeks after surgical dotted boxes indicate region of interest (ROI), representing bone distracted gaps (*n* = 3 per group); **(B)** (b). Quantification of bone tissue volume/total tissue volume and (BV/TV) insides bone distracted regions **(C)**. Evaluation of angiogenesis within the distracted gaps at 4 weeks after surgery **(C)** (a). Representative angiographs of the distracted gaps in the five groups (*n* = 3 per group) **(C)** (b). Quantification of vessel volume within the distracted regions (yellow dotted boxes indicate region of interest (ROI), representing bone distracted gaps). The data are expressed as the mean ± SEM of three independent experiments. *p < 0.05, compared with the control group; ^#^ *p* < 0.05, compared with the model group; ^Δ^ *p* < 0.05, compared the difference of the medium dose with the low and high doses in the TFDR group.

**FIGURE 7:**
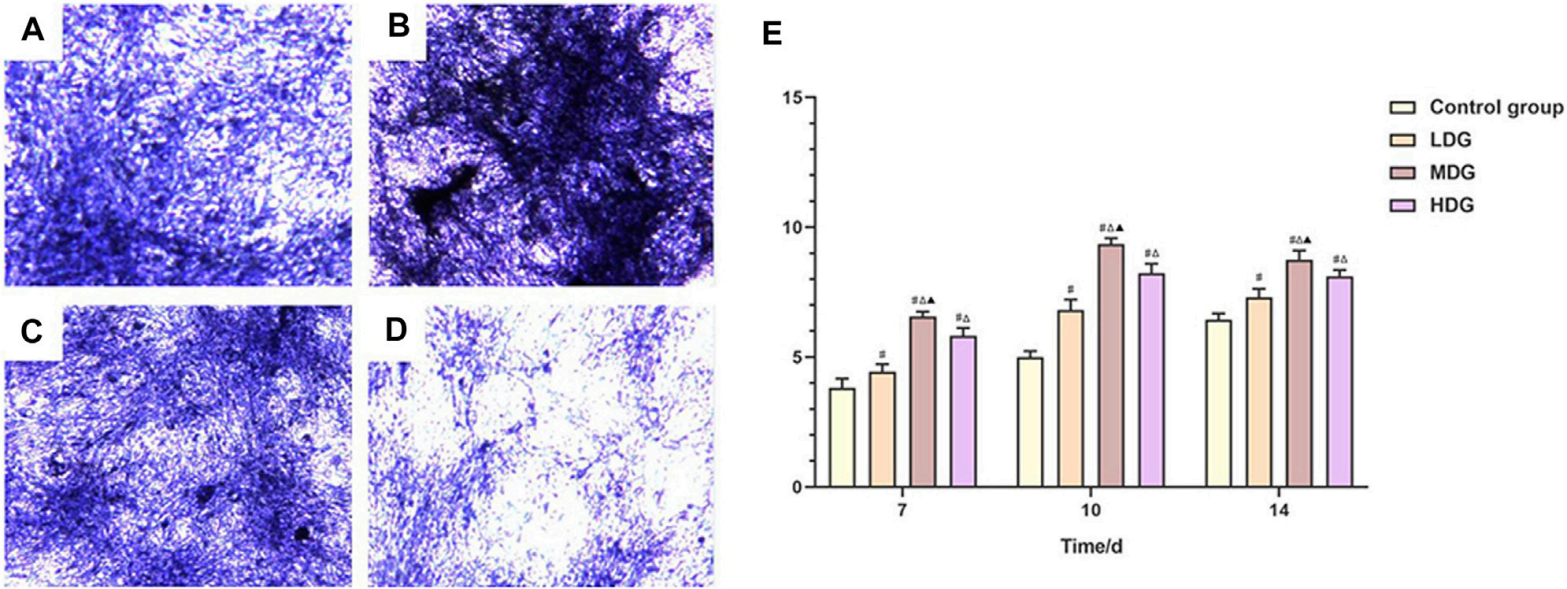
The ALP staining assay is performed to evaluate ALP activity after incubated for 10 days **(A)**. TFDR low dosage group; **(B)**. TFDR medium dosage group; **(C)**. TFDR high dosage group; control group; **(D)**. control group **(E)**. The ALP activity at each time point of the control group, TFDR low dosage group, TFDR medium dosage group, and TFDR high dosage group. The data are expressed as the mean ± SEM of three independent experiments. ^#^ *p* < 0.05 vs. control group, ^Δ^ *p* < 0.05 vs. TFDR low dosage group, ^▲^ *p* < 0.05 vs. TFDR high dosage group.

**FIGURE 9:**
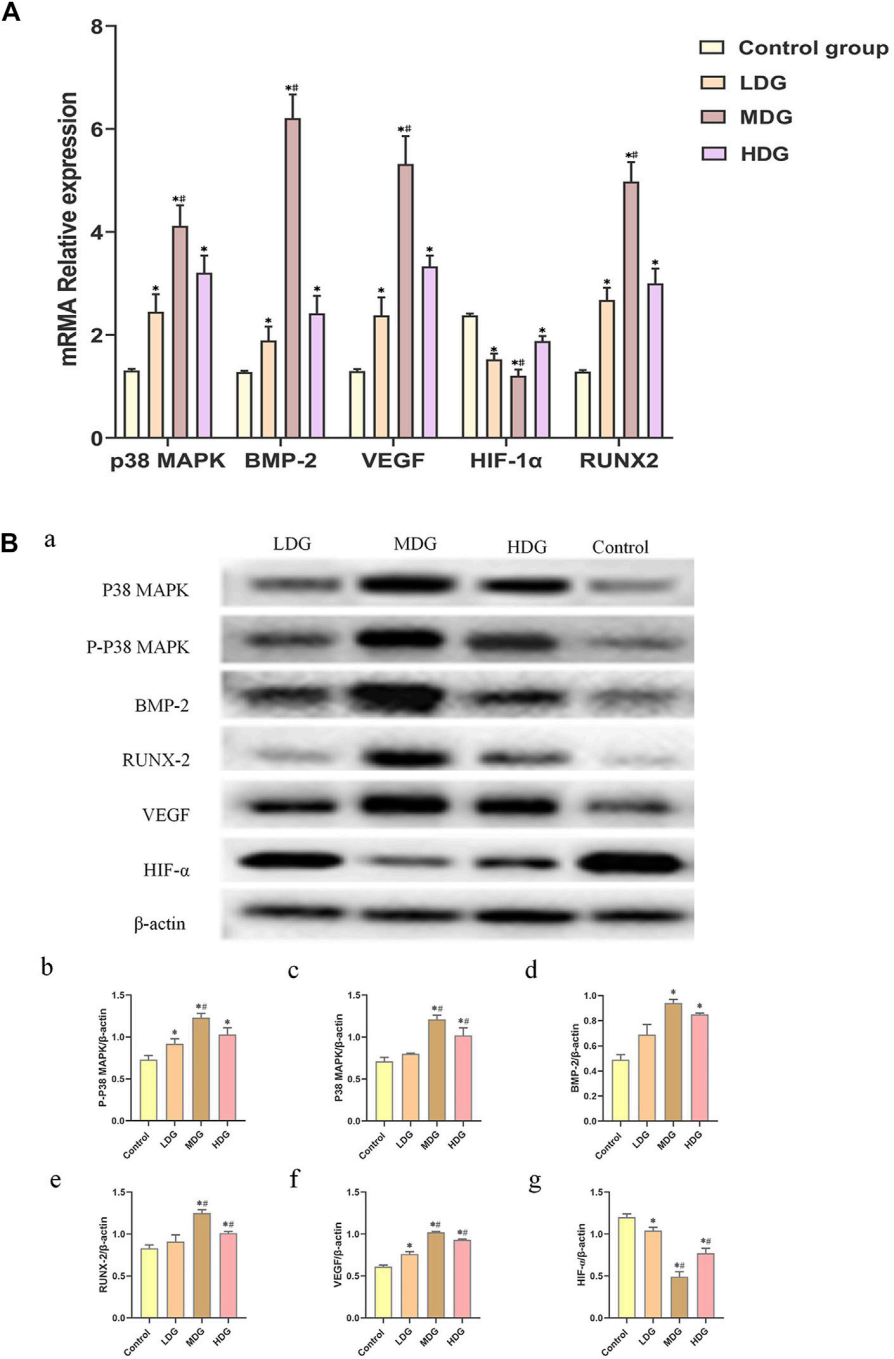
The expressions of p38 MAPK, BMP-2, VEGF, HIF-1α, and RUNX-2 mRNA on BMSCs by quantitative real-time PCR. The data are expressed as the mean ± SEM of three independent experiments. *p < 0.05 vs. control group, ^#^ *p* < 0.05 vs. the TFDR low dosage group, TFDR high dosage group. **(B)** (a) p38 MAPK, p-p38 MAPK, BMP-2, RUNX-2, VEGF, and HIF-1αprotein expression on BMSCs detected by western blot analysis. (b)–(g) were statistical analysis of (a). The data are expressed as the mean ± SEM of three independent experiments. *p < 0.01 vs. the Control group; ^#^ *p* < 0.01 vs. the TFDR low dosage group.

